# “Infostery” analysis of short molecular dynamics simulations identifies highly sensitive residues and predicts deleterious mutations

**DOI:** 10.1038/s41598-018-34508-2

**Published:** 2018-10-31

**Authors:** Yasaman Karami, Tristan Bitard-Feildel, Elodie Laine, Alessandra Carbone

**Affiliations:** 1 Sorbonne Université, CNRS, IBPS, Laboratoire de Biologie Computationnelle et Quantitative (LCQB), 75005 Paris, France; 2 Sorbonne Université, Institut des Sciences du Calcul et de des Données (ISCD), Paris, France; 3 Institut Universitaire de France (IUF), Paris, France

## Abstract

Characterizing a protein mutational landscape is a very challenging problem in Biology. Many disease-associated mutations do not seem to produce any effect on the global shape nor motions of the protein. Here, we use relatively short all-atom biomolecular simulations to predict mutational outcomes and we quantitatively assess the predictions on several hundreds of mutants. We perform simulations of the wild type and 175 mutants of PSD95’s third PDZ domain in complex with its cognate ligand. By recording residue displacements correlations and interactions, we identify “communication pathways” and quantify them to predict the severity of the mutations. Moreover, we show that by exploiting simulations of the wild type, one can detect 80% of the positions highly sensitive to mutations with a precision of 89%. Importantly, our analysis describes the role of these positions in the inter-residue communication and dynamical architecture of the complex. We assess our approach on three different systems using data from deep mutational scanning experiments and high-throughput exome sequencing. We refer to our analysis as “infostery”, from “info” - information - and “steric” - arrangement of residues in space. We provide a fully automated tool, COMMA2 (www.lcqb.upmc.fr/COMMA2), that can be used to guide medicinal research by selecting important positions/mutations.

## Introduction

The question of which and how amino acid sequence variations (re-)shape the conformational landscape of proteins and impact their function is one of outstanding importance in Biology. Disease-associated mutations can impair protein function in various ways, by destabilizing the protein structure, by shifting the equilibrium of conformation populations, or by modulating the binding affinity of the protein for its cellular partner(s), to name a few.

**Figure 1:**
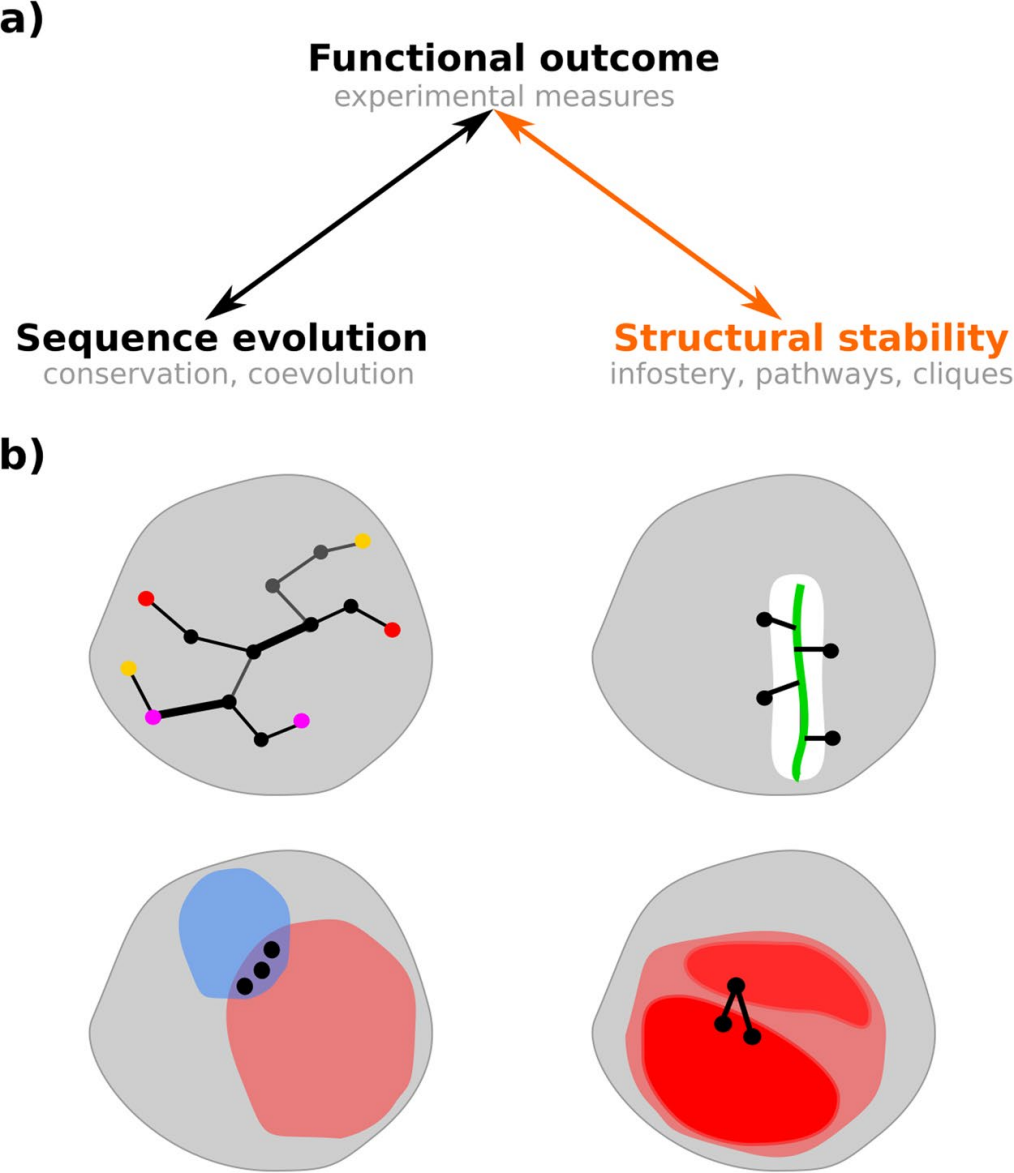
Sequence evolution-structural dynamics-function relationship and protein infostery. (**a**) Methods have been developed toward systematically assessing the link between the functional outcome of mutations and protein sequence evolution (arrow in black). Here, we investigate the link between functional outcome and protein structural dynamics (arrow in orange). (**b**) A protein is depicted as a grey shape and some residues are indicated by dots. Our approach relies on the identification of communication pathways (black edges between residues) and dynamical units (regions of the protein colored in red and blue). Top left: 3 overlapping communication pathways. The first and last residues of each pathway are colored the same way (yellow, red and magenta). Top right: 4 protein residues in direct communication with the protein’s ligand (green thick segment). Bottom left: 3 residues belonging to different types of dynamical units. Bottom right: 2 pairs of residues bridging two sub-regions of a dynamical unit. The more pronounced color of the two subregions indicate that they contain many pathways (dense communication).

Recent biotechnological advances have opened the way to systematically estimating the functional consequences of single-point mutations at every position in a protein, through deep mutational scanning^1^. So far, such analysis has been conducted on less than twenty proteins (see^2^ for a list of proteins and associated experiments), including the third PDZ domain of the brain synaptic protein PSD-95 (PSD95^*pdz*3^)^3^ and the *β*-lactamase TEM-1^4,5^. These experiments have revealed that a relatively small number of positions in a protein are highly sensitive to mutations^3,4^: a substitution of the amino acid at any of these highly sensitive positions by almost any other amino acid produces a deleterious phenotype. They also have stimulated the development of sequence analysis based methods to predict mutational outcomes at large scale (Fig. 1a, black arrow), some of them being much more accurate than widely used methods combining sequence and structure information^2,6^.

Even though sequence based methods can yield very accurate predictions of mutational phenotypic outcomes, they cannot shed light on the molecular mechanisms underlying them. Structure based methods provide a way to do so, and many studies have investigated the global and/or local effect of mutations on protein thermodynamic stability, hydrogen bond network and conformational dynamics^7–22^. There are few reported cases where crystallized protein mutants provide clear insights on the effects of the mutations (*e*.*g*. p53 cancer mutations affecting the arginines in contact with DNA^21,22^). However, in the vast majority of cases, the global shape of the protein remains unchanged upon mutations, even when the latter result in deleterious phenotypes^9^. This is very well exemplified by PSD95^*pdz*3^: the crystallographic structures of several deleterious mutants were solved and are very similar to that of the wild type^23^. In this context, characterizing the dynamical behavior of the system may reveal internal dynamics changes associated to the mutations, and help assess and interpret their phenotypic outcomes.

Such characterization can be realized by all-atom molecular dynamics (MD), and there are several examples in the literature where MD simulations, even of only a few tens of nanoseconds, revealed conformational rearrangements upon mutations and brought valuable insights into the molecular mechanisms underlying mutational outcomes^7,8,10–13,16–20^. The time scales reachable by MD have largely increased and it is now possible to simulate a mutated system for several microseconds^15^. Nevertheless, simulating tens of mutants on such long time periods remains very costly and the complete description of a protein’s conformational landscape is still far beyond reach. Another drawback is that identifying the protein properties (inter-residue distance, inter-domain angle, local unfolding, solvent exposure ...) that should be recorded along the simulation to guide an automatic detection of mutational effects, usually demands an expert knowledge of the system under study. Even with such knowledge, it may be difficult to determine what matters or not. Ideally, one would like to find general properties relevant for the systematic assessment of mutational phenotypic outcomes and that can be monitored in a computationally tractable way.

In the present study, we investigate whether information can be extracted in an automated way – and without requiring expert knowledge on the studied system, from MD trajectories toward the characterization of protein mutational landscapes. We perform the first large scale assessment of this question by simulating several hundreds of mutants. Specifically, we exploit relatively short (tens of nanoseconds) MD trajectories generated around a functional conformational state of a protein or a protein complex. We extract residues non-covalent interactions, displacements correlations, distances and secondary structures to build a network representing the average behavior of the studied state. We show that by analyzing the properties of the network (Fig. 1b) and their changes upon perturbations (mutations), one can identify the positions highly sensitive to mutations and discriminate between neutral and deleterious substitutions. We refer to our analysis as “infostery”, as it extracts information from the 3D arrangement of residues. Infostery analysis is intended to detect subtle changes between different states (mutated versus wild-type) or between (single or pairs of) residues and their local environment.

The concept of infostery is inspired from previous contributions to understanding information transmission across protein structures and its relevance for protein functional dynamics^24–35^. In recent years, several methods have been developed to identify “communication routes”^36–48^, “dynamic domains”^36,49–55^ and/or critical allosteric residues^49,56^ in proteins in an automated way (see also methods reviewed in^57^). Most of them construct a graph representing the protein where the nodes are the residues and the edges are determined based on the strength of non-covalent interactions (hydrogen-bonds, hydrophobic contacts, salt bridges ...) and/or on correlations between residues displacements. The latter are inferred either from all-atom MD simulations, or from more coarse-grained and computationally efficient approaches like the Elastic Network Model (ENM), where residues close in 3D space are linked by springs. The constructed graph is then analyzed to extract paths and communities of residues. Residues identified in the paths and/or playing particular roles (*e*.*g*. hubs) in the communities have been shown to be important for the protein structural stability and allosteric regulation. However, the agreement of computationally identified paths/communities with experimental data has been mostly assessed qualitatively, and little agreement has been found between different computational approaches or simulations^57^.

Here, we provide the first quantitative assessment of the link between inter-residue “communication” inferred from conformational ensembles and experimentally characterized protein mutational landscape, by producing and analyzing results on several hundreds of mutations (Fig. 1a, in orange). One of the originalities of our approach is that it accounts for the experimentally demonstrated fact that protein residues communicate either through stable non-covalent interactions^24^ or via changes in their local atomic fluctuations^58^. This allows defining different types of *dynamical units* (Fig. 1b, patches colored in red and blue) within a protein or protein complex. Residues lying within the same dynamical unit either move together and are linked by non-covalent interactions (patches in red), or have concerted high atomic fluctuations and are close to each other (patches in blue). Moreover, we aim at detecting small changes in the protein dynamical behavior, rather than large movements, and changes distributed all over the protein structure, not between two specific distant protein sites. Our strategy relies on average quantities computed from the simulations, which can be used profitably to capture the relative behavior of single residues or residue pairs. These aspects also motivate the introduction of the concept of infostery, which is different from structural/internal dynamics and allostery.

**Figure 2:**
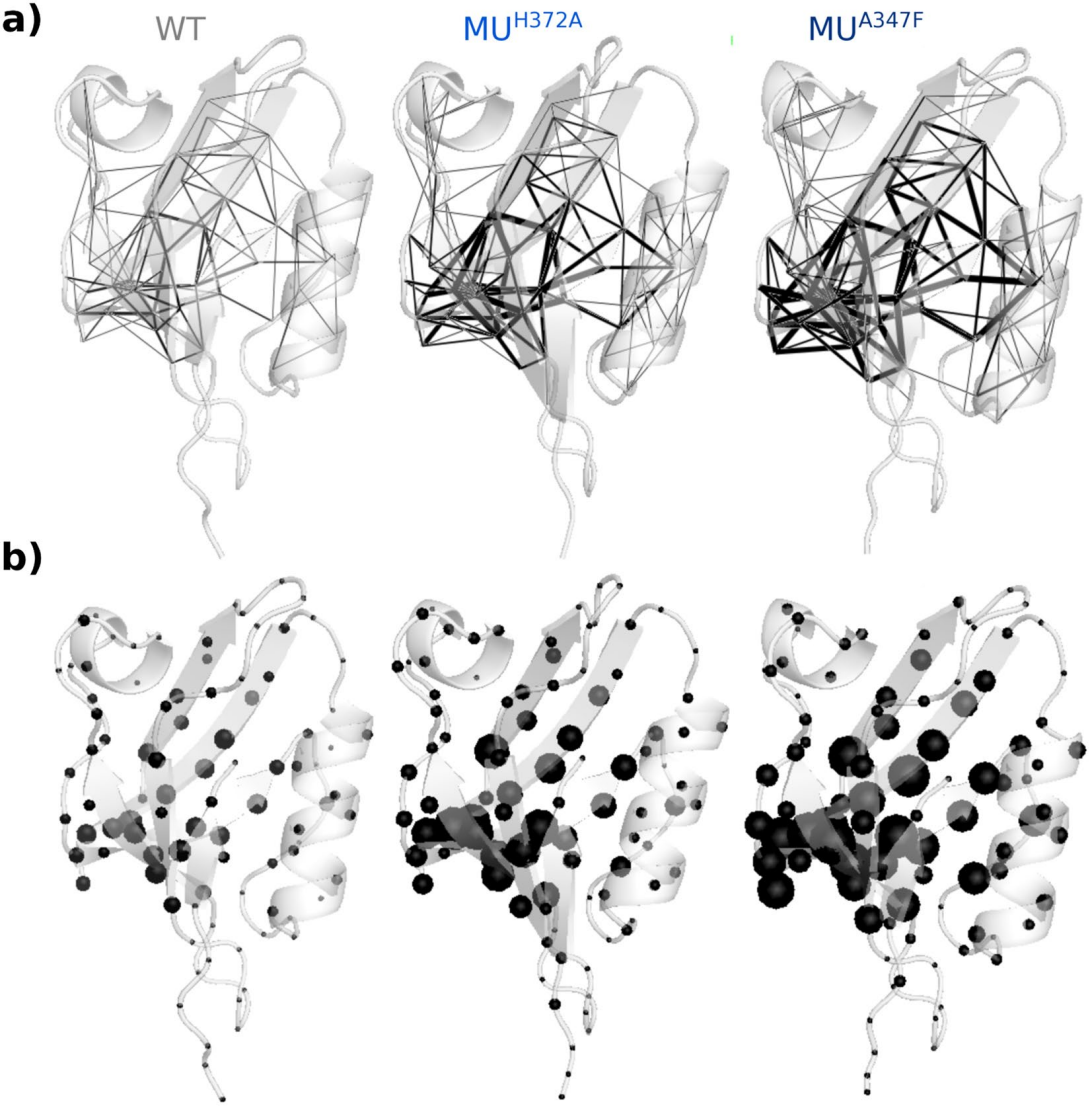
Infostery analysis of the wild-type PSD95^*pdz*3^-CRIPT peptide complex and two deleterious mutants. WT: wild-type. MU^*H*372*A*^: H372A mutant. MU^*A*347*F*^: A347F mutant. Pathway properties are mapped onto conformations averaged over 5 × 15 ns MD simulations. (**a**) Communication pathways (>3 residues) are displayed as segments linking residues’ C-*α* atoms. The thickness of each segment is proportional to the number of pathways linking the residue pair. (**b**) Pathway concentration is displayed as spheres centered on residues’ C-*α* atoms. The size of each sphere is proportional to the number of pathways crossing the residue.

We used the PSD95^*pdz*3^-CRIPT peptide complex as a test case. This choice was motivated by the availability of deep mutational scanning data^3^ measuring the changes in binding affinity of PSD95^*pdz*3^ for its cognate ligand (CRIPT peptide) upon every possible single mutations of the protein. This phenotype likely reflects the stability of the complex, which can be probed by MD simulations. In addition, a crystallographic study showed that highly deleterious mutants of the complex do fold into tertiary structures similar to that of the wild type^23^. We report the infostery analysis of the wild-type complex and of 175 mutants. To conduct our analysis, we generated conformational ensembles for the 176 systems by MD simulations in explicit solvent, totaling 17.6 *μ*s. We show that the deleterious mutants adopt the same structural shape as the wild type in solution and seem to behave the same. We further demonstrate that extracting *communication pathways* linking protein residues (Fig. 1b, top left, and Fig. 2) and quantifying them allow discrimination of the deleterious substitutions from the neutral or beneficial (gain-of-function) ones. Our results are statistically significant at large scale. Moreover, we show that the wild-type complex contains enough information to identify most of the positions highly sensitive to mutations. We obtain predictive performance similar to or higher than other sequence- or structure-based methods. The advantage of infostery is that it describes the structural roles of the highly sensitive positions, beyond their identification. We pinned down three general criteria to detect and characterize them: (1) stabilization of the binding of a ligand/partner by establishing direct communication with it (Fig. 1b, top right), (2) critical contribution to the protein structural stability by bridging independent secondary structure elements (Fig. 1b, bottom right), and (3) dual role in its dynamical architecture by being involved in two different types of communication (Fig. 1b, bottom left). These criteria can be summarized by the notion of “communication bridges”, either between the protein and the ligand, between regions of “dense” communication or between dynamical units of different types. Mutating residues that form these bridges may result in their breaking or weakening and impair the structural stability of the system.

We also applied our approach to two other systems, the *β*-lactamase TEM-1 and the complex between growth hormone (GH) and its receptors (GHR). Noticeably, the experimental data available for these systems, collected from deep mutational scanning or deep exome sequencing, do not directly reflect their stability (see *Discussion*). Nevertheless, we show that infostery analysis still provides information relevant to their mutational landscapes and we confirm that the residues identified by the three above mentioned criteria tend to be highly sensitive to mutations.

Our results indicate that the mutational landscape of a system can be characterized through infostery analysis, even when the mutational effects are not obvious by looking at the shape and motions of the protein. They contribute to answer to the questions ‘which’ and ‘how’ mutations affect protein structural stability. Specifically, our notion of communication provides a unique way to decipher protein structures and to determine which of their many non-covalent interactions are key players in maintaining their stability and function. Infostery analysis is implemented as a fully automated program, COMMA2, available at: www.lcqb.upmc.fr/COMMA2.

## Results

In this work, we propose new notions and measures associated to the concept of infostery and useful to describe the 3D arrangement of residues in conformational ensembles, and apply them to MD trajectories. We define a *communication pathway* as a chain of residues, where all the residues “communicate” efficiently with each other and any pair of residues adjacent in the pathway are linked by stable non-covalent interactions (Fig. 1b, top left). *Communication efficiency* is computed from the MD simulations as the inter-residue distance variance (Eq. 1), so that residues that move together (small variance) will be considered to communicate efficiently (see *Materials and Methods*). Two residues adjacent in a pathway are said to be in *direct communication*, as opposed to *indirect communication* when the residues are in the same pathway but not adjacent in it. The notion of direct communication is more refined than that of physical contact and should not be confounded with it: accounting for inter-residue displacements correlations enables discriminating among physical contacts. We define *dynamical units* as protein regions displaying particular properties: (*i*) a *pathway-based unit* (Fig. 1b, in red) is a set of residues linked by communication pathways, by transitivity, while (*ii*) a *clique-based unit* (Fig. 1b, in blue) is a set of proximal residues (in 3D space) with high concerted atomic fluctuations. Intuitively, residues in the former move together in a rather rigid way, while residues in the latter are more flexible. We define *communication bridges* as individual residues shared by different types of dynamical units (Fig. 1b, bottom left), or as pairs of residues linking the protein and the ligand (Fig. 1b, top right) or sub-regions within a pathway-based unit (Fig. 1b, bottom right) through direct communications. See below and *Materials and Methods* for more complete definitions.

In the following, we address two questions: (*i*) Is a particular substitution at a given position deleterious? (*ii*) What are the positions highly sensitive to mutations? To answer to (*i*), we exploit MD trajectories of 175 mutants of the PSD95^*pdz*3^-CRIPT peptide complex (complete list given in Supplementary Table S1) and compare them to the wild-type form. To answer to (*ii*), we characterize the infostery of the wild-type complex only. Then we compare our results to those obtained with structure- and/or sequence-based methods, and extend our analysis to two other systems, TEM-1 and the GH-GHR complex.

### PSD95^*pdz*3^-CRIPT peptide complex shape and motions

We simulated the dynamical behavior of the complex between PSD95^*pdz*3^ (residues 301 to 415) and the C-terminal CRIPT peptide (TKNYKQTSV, residues -8 to 0, see Supplementary Fig. S1a) in explicit solvent. We studied the wild-type form and 175 mutants, spanning 13 positions in the protein (see *Materials and Methods*). Each system was simulated for 100 ns (5 replicates of 20 ns), leading to a total of 17.6 *μ*s. The average structures computed from the MD simulations of the wild-type and mutated complexes look very similar (Fig. 2, see averaged conformations in cartoon, and Supplementary Fig. S2a). Moreover, the mutants display rather low RMS deviations (average values between 1 and 4 Å, Supplementary Fig. S3a) and low RMS fluctuations (median values between 0.6 and 1 Å, Supplementary Fig. S3b). The secondary structures also remain stable along the simulations. Consequently, the global shape and dynamical behavior of the complex seem unaltered by the mutations on the time scale of a hundred of nanoseconds.

### Increased pathway concentration in deleterious mutants

**Figure 3:**
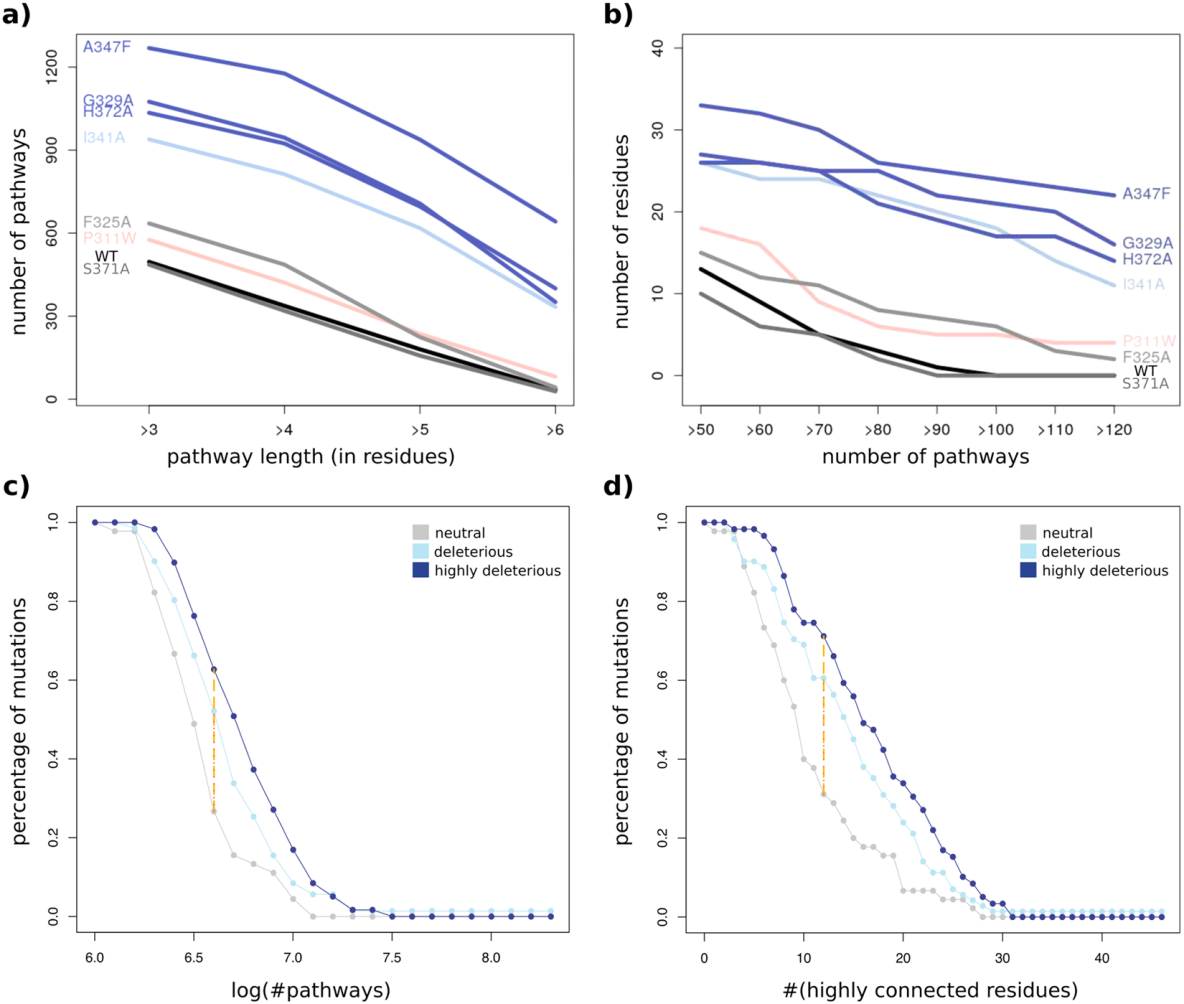
Effect of single-point mutations on pathway concentration in PSD95^*pdz*3^-CRIPT peptide complex. (**a**) Number of pathways longer than 3, 4, 5 or 6 residues. (**b**) Number of residues crossed by >50 to >120 pathways. The curves are colored according to the experimentally measured effects of the mutations: beneficial in pink, neutral in grey tones and deleterious in blue tones. (**c**,**d**) Inverse cumulative distribution functions of the number of pathways (>3 residue long) (**c**) and of the number of highly connected residues (>70 pathways) (**d**) for 175 mutations: 45 neutral (in grey), 71 deleterious (in light blue) and 59 highly deleterious (in dark blue). Each *y* value corresponds to the percentage of neutral, deleterious or highly deleterious mutations displaying a number of pathways (log) or a number of highly connected residues higher than the *x* value. The orange and red lines (superimposed on the plots) indicate the largest differences between the grey and dark blue curves and between the grey and light blue curves, respectively.

Communication pathways between residues were extracted from the MD trajectories (see *Materials and Methods*). To estimate the overall communication of the wild-type complex and of the mutants, we computed the number of pathways longer than 3, 4, 5 or 6 residues (Fig. 3a,c) and the number of residues crossed by >60 up to >120 pathways (Fig. 3b,d). We first illustrate the results on a subset of 7 mutations spanning different locations in PSD95^*pdz*3^ (Supplementary Fig. S4) and inducing different experimentally measured phenotypic outcomes^3^: P311W (beneficial), S371A and F325A (neutral), I341A (deleterious), H372A, G329A and A347F (highly deleterious). We observe a very sharp increase in the number and length of pathways (Fig. 3a) and of residues crossed by many pathways (Fig. 3b) in the deleterious mutants (shades of blue) compared to the neutral ones (shades of grey), the beneficial one (pink) and the wild type. See Fig. 2, Supplementary Fig. S2b,c for a visualization of the mapping of this information on the averaged MD conformations of the complex. These differences are not revealed by computing the volume of the convex hull defined by the network of pathways (>3 residue long) in each system (Supplementary Table S2).

Does this observation hold on a much larger set of mutations? The 175 studied mutations (Supplementary Table S1) were classified based on the experimental values reported in^3^ as neutral (45 mutations), deleterious (71) and highly deleterious (59) (see *Materials and Methods*). The distribution of the total number of pathways (>3 residue long) is significantly shifted to higher values for the highly deleterious mutations (Fig. 3c and Supplementary Fig. S5a–c, in dark blue) compared to the neutral mutations (in grey), while the deleterious ones display intermediate values (in light blue). The same observation can be made when looking at the number of *highly connected residues* (crossed by >70 pathways, Fig. 3d and Supplementary Figure S6a–c). To assess the statistical significance of the differences between the curves, we randomly permuted the mutations’ labels (neutral, deleterious or highly deleterious), determined the curves associated to the new labels and computed the biggest differences between the curves. We counted the number of times the differences between the random curves were bigger than those actually observed (Fig. 3c,d, red and orange segments). We found that the differences between highly deleterious (dark blue) and neutral (grey) mutations are statistically significant with p-values of 4*e* − 04 for the number of pathways and 2*e* − 04 for the number of highly connected residues. The differences between deleterious (light blue) and neutral (grey) mutations are significant at p-values of 0.0067 and 0.0035. Consequently, our results reveal a clear and statistically significant correlation, at large scale, between mutational phenotypic outcome and pathway concentration. The signal is sharper for the number of highly connected residues compared to the number of pathways.

Can we predict whether a particular substitution at a given position is deleterious or not? We tested whether we could single out the 59 highly deleterious mutations and discriminate them from the 45 neutral mutations, by applying a selection criterion based on the number of highly connected residues. Mutations leading to *x* times more highly connected residues than in the wild type, where *x* varies between 1 and 2.4, were predicted as highly deleterious (Table 1). The best Matthews correlation coefficient (MCC = 40%) is obtained with *x* = 2.2 (Table 1): 71% of the highly deleterious mutations are detected with a precision of 75%.

**Table 1:** Performance of the number of highly connected residues as predictors for experimental mutational outcome.

Coef	All (45 neutral + 59 highly del.)						Filtered (15 neutral + 41 highly del.)					
	Sens	Spe	Pre	Acc	F1	MCC	Sens	Spe	Pre	Acc	F1	MCC
1	97	27	63	66	77	34	95	40	81	80	88	44
**1**.**2**	93	31	64	66	76	32	**93**	**47**	**83**	**80**	**87**	**45**
1.4	86	40	65	66	74	30	85	47	81	75	83	34
1.6	78	47	66	64	71	26	73	60	83	70	78	31
1.8	75	60	71	68	73	35	71	67	85	70	77	34
2.0	75	62	72	69	73	37	71	67	85	70	77	34
**2**.**2**	**71**	**69**	**75**	**70**	**73**	**40**	66	67	84	66	74	29
2.4	66	71	75	68	70	37	61	67	83	62	70	25

The values of sensitivity (Sens), specificity (Spe), precision (Pre), accuracy (Acc), F1-score (F1) and Matthews correlation coefficient (MCC) are reported for different threshold values. The substitutions predicted as highly deleterious are those displaying a number of highly connected residues ${n}_{res} > x\ast {n}_{res}^{WT}$, where *x* is the coefficient reported in the first column of the table and ${n}_{res}^{WT}$ is the value computed for the wild-type complex. The “Filtered” set comprises only neutral mutations occurring frequently in homologous sequences and highly deleterious mutations occurring rarely or never. For each set of mutations, the line displaying the best MCC is highlighted in bold.

The experimental data^3^ contain some noise (Δ*E* values between −0.17 and 0.18 kcal/mol for the wild-type amino acids) that could impact our performance. To deal with this issue, we filtered out the mutations that were highly deleterious but occurring in a non-negligible number of homologous sequences and those that were neutral but occurring very rarely or never (see *Materials and Methods*). The reduced set comprises 15 neutral mutations and 41 highly deleterious ones. The distinction between the distributions for neutral and highly deleterious mutations are significantly improved on this set (Supplementary Figs S5d,e and S6d,e). The best MCC is of 45% and is obtained with *x* = 1.2 (Table 1): 93% of the deleterious mutations are detected with 83% precision. These results are robust over 500 different subsets of mutations of randomly chosen lengths and preserving on average the ratio between numbers of neutral and of deleterious mutations (Supplementary Fig. S7a). Moreover, on 500 balanced sets of 15 highly deleterious mutations and 15 neutral ones, our approach yields an average MCC of 47% (Supplementary Fig. S7b). Consequently, our infostery based approach proved efficient to discriminate highly deleterious mutations from neutral ones, by exploiting the fact that the former induce a bigger increase of the number of highly connected residues compared to the latter. We recommend to use the discriminative threshold of 1.2 with respect to the wild-type value.

### Prediction of highly sensitive positions from wild-type complex infostery

Here, we focus on the infostery of the wild type PSD95^*pdz*3^-CRIPT peptide complex to identify residues that serve as communication bridges within the complex. Our hypothesis is that these residues should be important for the stability of the complex and thus should significantly overlap with the set of 20 positions experimentally identified as highly sensitive to mutations (see *Materials and Methods*). We restricted our analysis to residues buried within the structure of the complex (see *Materials and Methods*), as residues exposed to the solvent may be relevant for interactions with other partners, for which we do not have any experimental data. We considered three different strategies that are explained below and whose predictive power is resumed in Table 2. Each strategy yields a set of deleterious positions and the three sets are rather complementary.

**Table 2:** Detection of highly sensitive positions in the PSD95^*pdz*3^-CRIPT peptide complex by infostery analysis of the wild-type form.

Strategy	Sens	PPV	Spe	Acc	True positives	False positives
path- and clique-based units^a^	25	100	100	82	G324, I341, H372, A376, L379	
direct communication w. ligand^b^	15	75	98	78	F325, I327, H372	N326
isolated direct communication^c^	65	93	98	90	L323, I327, G329, G330, I336, I341, A347, L353, V362, L367, H372, A375, L379	G356
all criteria (20 ns)	**80**	**89**	**97**	**93**	L323, G324, F325, I327, G329, G330, I336, I341, A347, L353, V362, L367, H372, A375, A376, L379	N326, G356
all criteria (50 ns)	85	89	97	94	L323, G324, F325, I327, G329, G330, I336, I338, I341, A347, L353, V362, L367, H372, A375, A376, L379	N326, I337

The performance values, sensitivity (*Sens*), precision or positive predictive value (*PPV*), specificity (*Spe*) and accuracy (*Acc*), are given in percentages. They are computed for the set of 20 highly sensitive positions given in *Materials and Methods*. ^a^Residues detected in both a pathway-based dynamical unit and a clique-based dynamical unit with very high confidence. ^b^Residues forming direct communications with the ligand. ^c^Residues forming isolated direct communications between them (see *Materials and Methods*). The three first lines correspond to the analysis of 5 replicates of 20 ns, while the last line corresponds to the analysis of the 5 replicates extended to 50 ns.

The first strategy extracted residues bridging two dynamical units of different types (Fig. 1b, bottom left). In the complex, 2 pathway-based units (Supplementary Fig. S7, in red and in pink) and 4 clique-based units (Supplementary Fig. S7, in blue tones) were detected. Owing to their different properties (see *Materials and Methods*), the two types of units share a small number of residues in common, namely 5. These residues are crossed by few small pathways (≤4 residues) and display relatively low atomic fluctuations (compared to the residues belonging only to a clique-based unit). All of them are highly sensitive to mutations, representing 25% of the set (Table 2).

**Figure 4:**
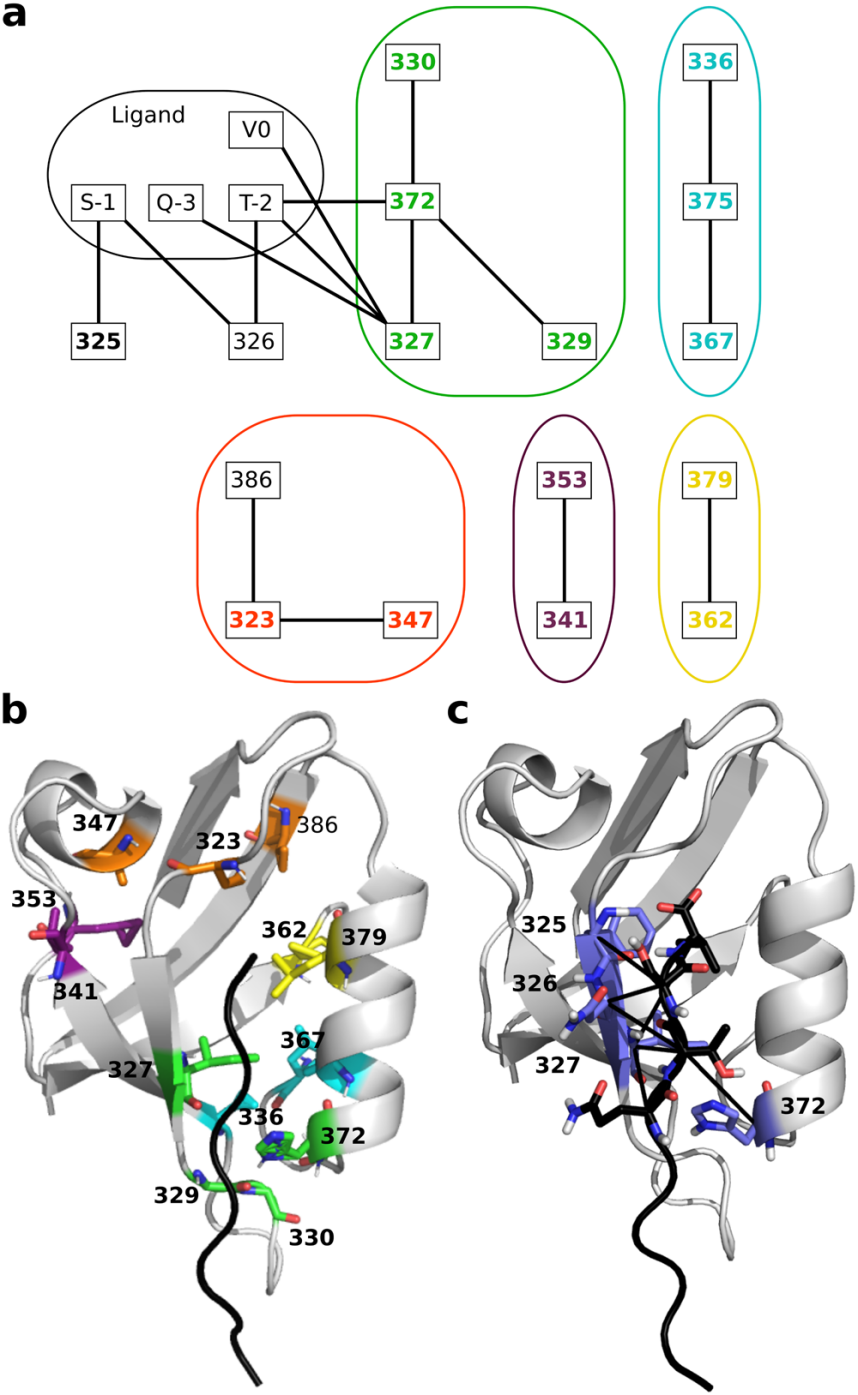
Network of residues in direct communication in wild-type PSD95^*pdz*3^-CRIPT peptide complex. (**a**) Each node corresponds to a residue and each edge corresponds to a direct communication, detected either as isolated within the PDZ domain, or between PDZ and its ligand. Residues in bold are deleterious hotspots. The connected components extracted from the subnetwork where the nodes and edges associated to the ligand are removed are encircled in different colors. (**b**) The residues involved in communications within PDZ are shown as sticks and colored according to the connected component to which they belong. (**c**) The residues from the ligand (in black) and from PDZ (in slate) in direct communication are shown as sticks. The communications are displayed as black lines.

The second strategy extracted residues bridging the protein and the ligand (Fig. 1b, top right). Four protein residues were found in direct communication with residues from the ligand (Fig. 4a,c). They represent 15% of the set at a precision of 75% (Table 2). Let us recall that the notion of direct communication implies physical contact and efficient communication (see *Materials and Methods*). Using only the physical contact criterion leads to 13 residues, representing 40% of the highly sensitive positions but with a lower precision (61%).

**Figure 5:**
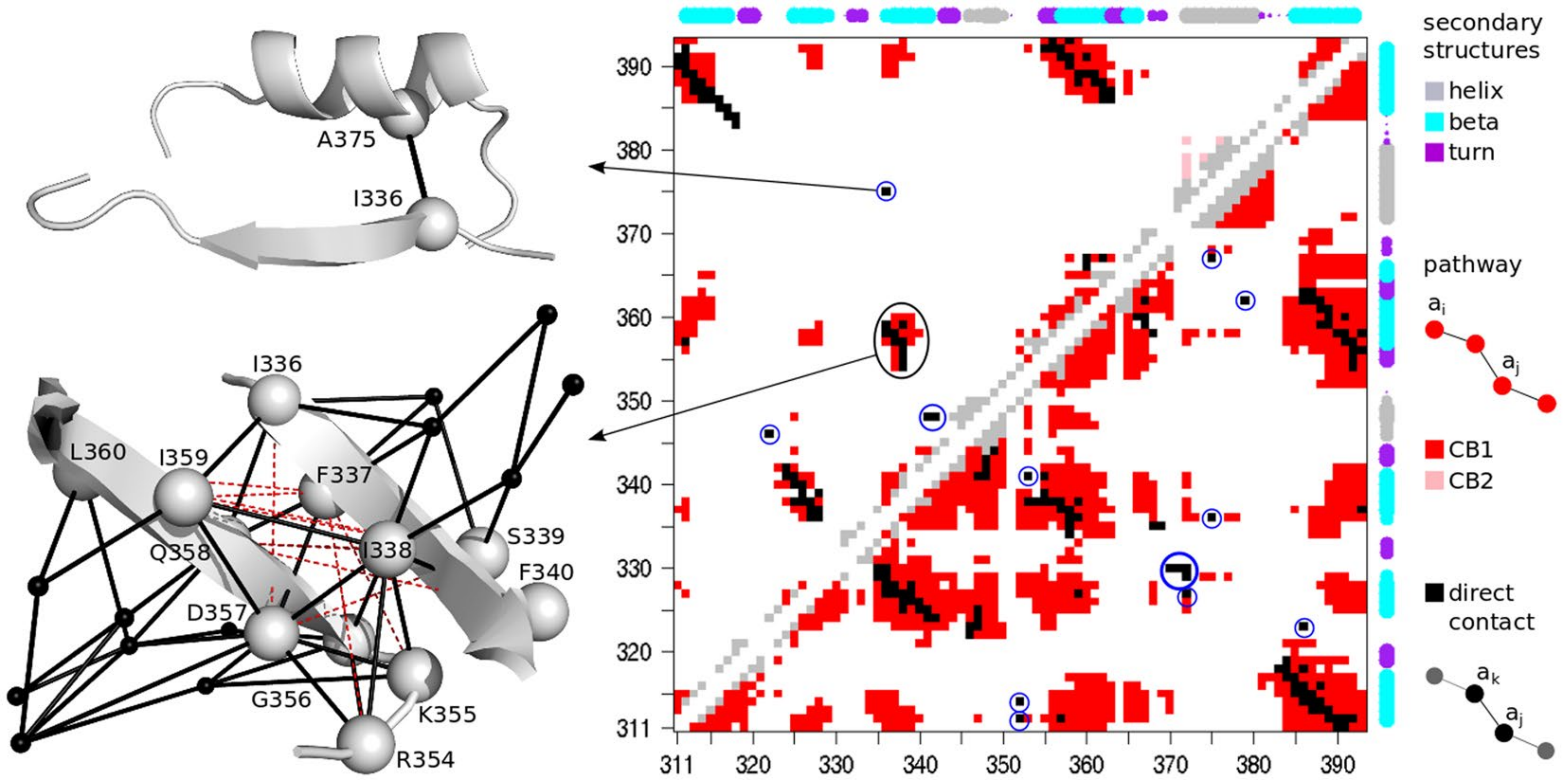
Dotplot representing direct and indirect communication between PSD95^*pdz*3^ residues. Upper triangle: default communication propensity threshold. Lower triangle: threshold corresponding to 65% quantile of the communication propensity distribution. Each dot stands for the existence of a communication pathway linking the 2 residues indicated in x and y-axis. If the 2 residues are less than 4 residues away in the protein sequence, the dot is colored in grey. Otherwise, if the 2 residues are adjacent in a pathway (direct communication), the dot is in black. If they are not adjacent (indirect communication), the dot is colored according to the pathway-based unit to which the residues belong (red or pink, same color code as in Supplementary Fig. S7, on the left). Isolated direct communications are encircled in blue. The secondary structures are also indicated (size of the rounds proportional to the persistence of the secondary structure along the MD trajectories). On the left, two communication motifs are mapped onto the 3D structure of PDZ, represented as a cartoon. The pathways (>3 residues) linking the residues in the motifs are displayed as black solid lines. The C-*α* atoms of the residues belonging to the motif are represented as grey spheres (black smaller spheres outside the motif). Dashed red lines indicate indirect communications.

The third strategy extracted residues bridging different sub-regions of pathway-based dynamical units (Fig. 1b, bottom right). The intuition here is to identify pairs of residues whose communication signal is strong compared to the residues around them, so that disrupting these pairs should have an impact on the overall communication of the unit. Specifically, we extracted pairs of residues that were (1) far away in the sequence, (2) located in the same dynamical unit, (3) in direct communication and (4) isolated (see *Materials and Methods*). On the dot plots displaying all (direct and indirect) communications (Fig. 5), one can observe that most direct communications between residues far away in the sequence (black dots) are grouped together and surrounded by indirect ones (colored dots). This indicates that the residues surrounding them are also in direct communication between each other, or indirectly linked by communication pathways. Yet, there are a few direct communications that appear isolated in the plot (isolated black dots, encircled in blue, see *Materials and Methods*). They correspond to residue pairs that form communication bridges between two protein segments while the other residues from the two segments communicate with significantly poorer efficiency (Fig. 5, upper left cartoon, and Fig. 1b, bottom right). Direct and indirect communications are determined by setting a communication propensity threshold (see *Materials and Methods*). At the default threshold value (Fig. 5, upper left triangle), 4% of all residue pairs far away in the sequence establish communications (Supplementary Table S3): 203 are indirect and 70 are direct, among which 4 are isolated (Fig. 5, black dots encircled in blue). Upon increasing the threshold, the communication patterns change and new isolated black dots progressively appear on the plot (Supplementary Fig. S8). At the maximal threshold value (Fig. 5, lower right triangle), 16% of all residue pairs establish 896 indirect and 109 direct communications (Supplementary Table S3), among which 12 are isolated. By gradually increasing the threshold from default to maximal value (see *Materials and Methods* and Supplementary Fig. S8) and filtering out residues exposed to the solvent, we identified 9 isolated direct communications involving 14 residues (Fig. 4a,b). They form a network comprised of 5 connected components (Fig. 4a), each component encompassing several secondary structure elements remote from each other in the primary sequence (Fig. 4b). All isolated direct communications are found between different secondary structure elements. Except for one, all detected residues were identified as highly sensitive to mutations in^3^. Consequently, this strategy retrieves 65% of the highly sensitive positions with a precision of 93% (Table 2).

This analysis demonstrated that by exploiting short MD simulations of only one conformational state of the wild-type PSD95^*pdz*3^-CRIPT peptide complex, without any insight into the conformational changes induced by any mutation, we could predict 80% of the highly sensitive positions with a precision of 89% (Table 2). Importantly, our analysis enables describing the role of these positions in the inter-residue communication and dynamical architecture of the complex.

### Robustness of the results

To assess the robustness of our results with respect to simulation length, we extended each of the 5 MD simulation replicates of the wild-type PSD95^*pdz*3^-CRIPT peptide complex to 50 ns (2.5 times longer than the initial 20 ns). We applied the three analyses described above to the extended simulations. The resulting list of predicted sensitive positions is very similar to that obtained with the 20-ns replicates (Table 2). Specifically, G324, I341 and H372 are not detected anymore as residues bridging clique- and pathway-based units (first strategy) but they are still detected as forming isolated direct communications (third strategy). A new true positive, I338, and a new false positive, F337, are detected as forming isolated direct communications, while the false positive G356 is not detected anymore. Overall, 85% of the highly sensitive positions were identified with a precision of 89%. Consequently, our infostery analysis is robust to variations in simulation length.

### Transferability to other systems

We extended our analysis to the *β*-lactamase TEM-1 (Supplementary Fig. S1b) and the complex between growth hormone (GH) and its receptor (GHR, Supplementary Fig. S1c). These two systems are much bigger than the PSD95^*pdz*3^-CRIPT peptide complex (Supplementary Table S4) and they adopt completely different folds (Supplementary Fig. S1). We generated MD trajectories for the wild type protein (TEM-1, 263 residues) or complex (GH-GHR, 569 residues) and applied our infostery analysis to detect residues forming communication bridges (1) between dynamical units of different types, (2) with the bound partner or (3) within a pathway-based dynamical unit (three strategies detailed above, see also *Materials and Methods*). In the case of TEM-1, only one of the three strategies (strategy 3) could be applied. Indeed, no ligand was included, so that we could not detect residues in direct communication with the ligand (strategy 2). Moreover, the protein remained very stable along the simulations (average RMSD of 1.81 ± 0.17 Å), with very small fluctuations (RMSF values between 0.4 and 1.9 Å), so that no clique-based dynamical unit was detected (strategy 1).

TEM-1 is an enzyme providing antibiotic resistance by binding to the antibiotic and breaking its structure. The effects of 95% of all possible amino-acid substitutions (19 × 263 = 4997) on the ability of the protein to confer antibiotic (ampicillin) resistance were measured experimentally^4^. From this experiment, a tolerance value *k*$\ast$ (between 1 and 20) was computed for each position, measuring the sensitivity of that position to mutations: a *k*$\ast$ value of 20 means that all 19 possible substitutions are neutral while a *k*$\ast$ value of 1 means that all substitutions are deleterious^4^. At one end of the spectrum, about 50% of the protein residues tolerate more than 15 substitutions ($k\ast  > 16$). At the other end, 8 positions (3%) tolerate less than 1.5 substitutions ($k\ast  < 2.5$) and are identified as highly deleterious in^4^. Note that this definition is much more stringent than that used for PSD95^*pdz*3^ ^3^, where the highly deleterious positions represent 24% of the protein (20/83). Six of these 8 positions are detected by our infostery analysis: S70, K73, D131, E166, D179, T181 and K234. Our inability to retrieve 2 residues, S130 and G251, can be explained by the following observations: S130 is directly involved in catalysis^4^ and in direct contact with the ligand (PDB code: 1M40^59^), which is absent in our simulations; G251 is exposed to the solvent, suggesting a role in the interaction with a protein partner. In total, we identified 34 residues (13% of the protein) forming a network of isolated communication bridges (Supplementary Fig. S9, in spheres) comprised of 5 connected components (indicated by the colors of the links). This network contains all but one (S130) of the 9 residues known to be part of the catalytic cleft (Supplementary Table S5). As observed for PSD95^*pdz*3^, all the detected bridges link different secondary structure elements (Supplementary Fig. S9). The network comprises half of the positions tolerating less than 4 substitutions (Supplementary Table S6, $k\ast \le 5$). Moreover, 79% of the residues in the network tolerate less than 9 substitutions (Supplementary Table S6, $k\ast \le 10$). These results confirm the power of our infostery analysis to identify positions sensitive or highly sensitive to mutations.

GH is a peptide hormone, folded as a four-helix bundle (184 residues), that stimulates growth by binding to two monomers of its receptor GHR (Supplementary Fig. S1c). Systematic experimental measurements of mutational phenotypic outcomes are not available for this system. Nevertheless, one can exploit data from homologous sequence analysis across different species, and from high-throughput exome sequencing of human individuals (Exome Aggregation Consortium database^60^) to define a set of 30 positions likely intolerant to substitutions (see *Materials and Methods*). Among them, 21 positions (70%) were detected by our infostery analysis (Supplementary Fig. S10, in red, and Supplementary Table S7) and most of those positions form isolated direct communication within the protein (Supplementary Fig. S10, strategy 3). The detection includes C53, C165 and T175 (Supplementary Fig. S10, indicated by stars, and Supplementary Fig. S10, in magenta), which were experimentally identified as crucial for the stability of the GH-GHR complex^61,62^. In total, our infostery analysis identified 45 positions, representing about one quarter of the protein. The increasing availability of experimental data on mutational effects will enable further assessment of the significance of all these positions.

### Comparison with other structure-based methods

We compared our results with those obtained from six other structure-based approaches (Table 3), implementing different protocols to generate conformational ensembles and different algorithms to extract biological information relevant to mutational outcome prediction and to highly sensitive positions identification. ENcoM (Elastic Network Contact Model)^63^, STRESS (STRucturally identified ESSential residues)^49^ and PRS-CG (Perturbation Response Scanning-Coarse-Grained)^40^ infer protein motions by modeling the protein as an Elastic Network Model (ENM), which is more coarse-grained and more computationally efficient than all-atom MD simulations. The RIP (Rotamerically Induced Perturbation) protocol^38^ mimics the side-chain motions sampled during MD simulations (without distorting backbone secondary structure)^40^. In terms of computational efficiency, it is intermediate between ENM and MD simulations. PRS-REMD (Perturbation Response Scanning-Replica Exchange Molecular Dynamics)^40^ and CARDS (Correlation of all Rotameric and Dynamical States)^56^ rely on all-atom MD simulations, and are consequently computationally equivalent to our approach.

**Table 3:** Predictive performance of other sequence- and structure-based methods.

Prediction of mutational outcomes								
Method/Strategy		Set of mutations	Sens	PPV	Spe	Acc	F1	MCC
Structural		All: 45 neu. + 59 highly del.	92	31	64	65	75	29
Dynamics	ENCoM^a^	Filtered: 15 neu. + 41 highly del.	88	33	78	73	83	24
**Detection of highly sensitive positions**								
**Method/Strategy**			**Sens**	**PPV**	**Spe**	**Acc**	**True positives**	
Structural Dynamics Analysis	STRESS^b^		25	33	84	70	I338, L353, V362, L367, A375	
	PRS-CG^c^		75	44	78	73	I327, I328, G329, G330, I336, I338, I341, A347, L353, I359, V362, L367, H372, A375, L379	
	PRS-REMD^d^		70	42	70	72	F325, I327, I328, G329, G330, I336, I338, I341, I359, V362, L367, A375, L379 I388	
	RIP^e^		50	56	87	81	L323, F325, I336, A347, L353, I359, V362, L367, A375, L379	
	CARDS^f^		45	36	75	67	L323, I327, I328, I338, I341, L353, L367, H372, L379	
Sequence Analysis	JET^g^ JET/surf^i^		85 85	65 81	86 94	86 92	L323, G324, F325, I327, G329, G330, I336, I338, A347, L353, I359, V362, H372, A375, A376, L379, I388	
	SCA^h^ SCA/surf^i^		75 75	75 94	92 98	88 93	L323, F325, I327, G329, G330, I336, A347, L353, I359, V362, H372, A375, A376, L379, I388	
	MST^h^ MST/surf^i^		80 80	64 80	86 94	84 90	L323, G324, I327, G329, G330, I336, I341, A347, L353, I359, V362, H372, A375, A376, L379, I388	
	DCA^h^ DCA/surf^i^		70 70	70 82	94 95	86 89	L323, G324, I327, G329, G330, I336, I338, A347, L353, I359, V362, H372, A375, L379	

The performance values, sensitivity (*Sens*), precision or positive predictive value (*PPV*), specificity (*Spe*) and accuracy (*Acc*), are given in percentages. On top, they are computed for two selected sets of mutants (“all” and “filtered”, compare with Table 1). At the bottom they are computed for the set of 20 highly sensitive positions given in *Materials and Methods* (compare with Table 2). ^a^Performance obtained from ΔΔ*G* values computed by combining Elastic Network Contact Model (ENCoM)^63^ and FoldX^91^, as described in^64^. Mutations predicted as highly deleterious are those with ΔΔ*G* > 0. ^b^Residues identified as interior-critical by STRucturally identified ESSential residues (STRESS)^49^. ^c^Residues identified by perturbation response scanning (PRS) using a coarse-grained model (elastic network model)^40^. ^d^Residues identified by perturbation response scanning (PRS) using all-atom restrained-replica exchange molecular dynamics (REMD)^40^. ^e^Residues identified as forming buried tertiary couplings, defined based on rotamerically induced perturbation (RIP)^38^. ^f^Residues displaying strong correlation between their rotameric states along MD simulations and those of all other residues in the protein, as computed by CARDS^56^. Residues in the top 30% of the distribution are considered. ^g^Highly conserved residues (see *Materials and Methods* for a definition of the conservation measure used here). ^h^Co-evolved residues detected by three different methods. ^i^Residues exposed to the solvent are not considered.

ENcoM directly predicts the effects of mutations on protein dynamics and thermostability^64^. Contrary to most ENM-relying methods, it accounts for the nature of amino acids, and that is why we chose it to classify our set of highly deleterious and neutral mutations in PSD95^*pdz*3^ (Table 3). ENcoM performance are lower than those obtained from our infostery analysis on both the complete (*All*) and filtered datasets (see Table 1 for comparison). The accuracy is lower by 7–8 points and the MCC by more than 10 points. The five other tested methods analyze correlations between residues displacements or dihedral angles, some of them also accounting for residue interactions, toward identifying residues critical for protein stability and/or information transmission (allosteric communication). We used them to predict the set of 20 positions highly sensitive to mutations in PSD95^*pdz*3^ (Table 3). STRESS and RIP identify residues buried inside the protein and forming strong couplings between modules/domains (STRESS) or secondary structure elements (RIP). They detect only 25% and 50% of the highly sensitive positions, with precisions of 33% and 56% respectively (Table 3). Their detections are largely less sensitive and less precise than our infostery analysis (Table 2). Let us recall that our complete infostery detection covers 80% of the highly sensitive positions with 89% precision. Even if we consider only isolated direct communications, whose definition is somewhat similar to the concepts used in the two tested methods, we achieve higher sensitivity (65%) at higher precision (93%, see Table 2). PRS detects 75% (resp. 70%) of the positions with a precision of 44% (resp. 42%) when applied to an ENM (PRS-CG) or MD simulations (PRS-REMD), respectively (Table 3). These statistical performances are significantly lower than those obtained with our infostery analysis (Table 2). Finally, CARDS identifies residues whose rotameric states are globally strongly correlated to those of all other residues in the protein. It detects a bit less than half of the highly sensitive positions (45%) with a precision of 36% from our MD trajectories (Table 3). Consequently, this method is less efficient in extracting highly sensitive positions than our infostery analysis (Table 2) when applied to the same data.

### Comparison with sequence-based methods

We also investigated the relationship between the signals captured by infostery analysis and those detected by sequence analysis on PSD95^*pdz*3^. First, we extracted 26 evolutionarily conserved positions using Joint Evolutionary Trees^65^ (see *Materials and Methods*). Among them, 17 are highly sensitive to mutations, yielding an accuracy of 86% (Table 3, JET). Second, we obtained co-evolved residues by using three different methods, namely Statistical Coupling Analysis (SCA)^66^, Direct-Coupling Analysis (DCA)^67^ and Maximal SubTrees (MST)^68^ (see *Materials and Methods*). SCA and DCA are statistical methods that infer couplings between residues from the alignment and require a large set of sequences. By contrast, MST relies on a combinatorial approach based on the analysis of the distance tree associated to the alignment, and on the combinatorics of the subtrees preserving conservation signals. The three methods display comparable accuracies, in the range 84–88% (Table 3). They detect as much as or slightly less highly sensitive positions, compared to our infostery analysis (Table 2), and with lower precision. The precision can be significantly improved (up to 94%) by filtering out the exposed residues (Table 3, /surf). This shows that combining signals extracted from sequence analysis with a very simple structure-based descriptor permits to precisely single out most highly sensitive positions. Noticeably, co-evolution signals do not bring significant new information compared to evolutionary conservation on this system (Table 3, JET/surf).

Overall, this analysis revealed a very good overlap between the set of infostery-detected residues, the set of conserved/coevolved and buried residues, and the set of residues highly sensitive to mutations. This clearly indicates a link between the evolutionary constraints and the structural constraints that apply to the PDZ domain to ensure/adapt its function. Our infostery analysis provides a physical interpretation of conservation/coevolution signals.

## Discussion

In this work, we have investigated the link between computationally characterized structural stability and experimentally measured mutational outcomes. We have introduced new measures and concepts to extract pertinent biological information from conformational ensembles in an automated way and have demonstrated their usefulness in predicting protein mutational landscapes.

We have generated conformational ensembles for the wild-type PSD95^*pdz*3^-CRIPT complex and for 175 mutants. This is, to our knowledge, the first study reporting MD simulations of protein mutants at such a large scale. Our simulations revealed that the mutants adopt a global shape similar to that of the wild type, and they seem to behave the same on the time scale of a hundred of nanoseconds. This is in agreement with recently published structures of the complex^23^: the mutants H372A (PDB code: 5HFB) and G330T (PDB code: 5HEY) almost perfectly superimpose on the wild-type complex (PDB codes: 1BE9, 5HEB) with RMSD values lower than 1 Å. This is also consistent with the observation that PSD95^*pdz*3^ is particularly stable among PDZ domains^40^. And this is one important reason that calls for the development of new measures capturing the differences between protein dynamical behaviors.

We used a network formalism to extract communication pathways from the simulations and revealed that pathway concentration is correlated with the severity of (experimentally measured) mutational outcome. The vast majority of mutants (153 over 175) display a number of highly connected residues (crossed by >70 pathways) higher than that of the wild type, and this effect is significantly more pronounced in deleterious mutants compared to neutral and beneficial ones. One may wonder how one can physically interpret this increase in pathway concentration. By definition, communication pathways link residues that communicate efficiently across the protein structure. We measure communication between two residues as the variance of their distance: the lower the variance the more efficient the communication (see *Materials and Methods*). The creation of a pathway between the two residues is conditioned by this variance being lower or equal to a reference value, the *communication propensity threshold*, which corresponds to a local average along the protein backbone (computed between every residue *i* and residues from *i* − 4 to *i* + 4, see *Materials and Methods*). Hence, communication efficiency across the protein structure is defined relatively to local communication efficiency along the backbone. In this context, the increase in pathway concentration can be due to more efficient communication between residues far away in the sequence (indicated by lower distance variances between these residues), or to less efficient local communication along the backbone (indicated by higher communication propensity threshold), or to a combination of both. In the mutants studied here, the increase in pathway concentration is correlated with less efficient local backbone communication (Supplementary Fig. S11).

Importantly, we have demonstrated that the wild-type complex contains all information necessary to identify most of the positions that ‘matter’ with very high precision. The predictive power of our approach is similar or higher than other structure-based or sequence-based methods. Compared to the latter, it has the drawback of being more computationally expensive, but the advantage of also describing the structural roles of crucial positions. Moreover, beyond the characterization of highly sensitive positions, infostery also provides a detailed description of the dynamical organization of the complex through the identification of dynamical units. We identified 2 pathway-based units matching the main secondary structure elements of the complex (Supplementary Fig. S7, in red and pink), and 4 clique-based units mainly comprised of loops (Supplementary Fig. S7, in blue tones). To interpret this decomposition, we compared our results with a recent study characterizing the mechanics of another PDZ domain, LNX2^*PDZ*2^, by electric-field stimulation^69^. By mapping LNX2^*PDZ*2^ residues to their counterparts in PSD95^*pdz*3^, we found that 75% of the residues displaying the highest electric-field induced displacements (>0.5 Å, see Fig. 5b in^69^) were detected in clique-based units by our analysis (Supplementary Fig. S7, in blue tones). Moreover, the directions of the displacements in the experiment agree with our decomposition into different clique-based units: residues belonging to the same unit move in the same direction, while directions are different between different units (compare Supplementary Fig. S7 with the arrows on Fig. 5a–c in^69^). Let us stress that electric-field induced displacements were shown to be functionally significant as they match ligand-induced displacements inferred from X-ray crystallographic structures of PDZ domains^69^. Consequently, our infostery analysis provides a way to capture functionally significant structural properties of the protein.

Extending our analysis to two other unrelated systems confirmed that positions sensitive to mutations tend to form communication bridges and that conservation and infostery-based signals significantly overlap. In these two cases, the experimental data used to validate our predictions are noticeably different from those used for PSD95^*pdz*3^. For TEM-1, we used deep mutational scanning data measuring the ability of the protein to target and degrade antibiotics. This phenotype implies protein stability, ability to bind to the target (antibiotic), to catalyze a chemical reaction (breaking the antibiotic) and to dissociate from the reaction products, whereas the phenotype measured for PSD95^*pdz*3^ reflected more directly the stability of the PSD95^*pdz*3^-CRIPT peptide complex. It is also worth mentioning that several studies have experimentally characterized TEM-1 mutational landscape^4,5,70,71^, and results reported in these studies only partially agree. For GH, we used exome sequencing data from human individuals, with the hypothesis that positions displaying no to very little variability in a large population of individuals are likely sensitive positions. We only expect an indirect link between this type of data and the stability of the GH-GHR complex. Yet, despite a lower precision, our approach is still able to pinpoint key positions in TEM-1 and GH, and describe their role in the structural stability of the proteins.

One key ingredient of our infostery analysis is the usage of relatively short (tens of ns) MD simulations. This ensures the applicability of the method at large scale, in a computationally tractable way. The simulation lengths for the three studied systems were chosen empirically and adjusted based on RMSD profiles. We aimed at obtaining sufficient sampling around a functional state of the wild-type protein or complex. We avoided sub-sampling and guaranteed robustness of the results by running several replicates, computing residue persistency scores and varying the communication propensity threshold (see *Materials and Methods*). Moreover, we assessed the robustness of our detection of highly sensitive positions in PSD95^*pdz*3^ with respect to simulation length variation of several tens of ns. Running much shorter simulations would probably lead to poor results because of sub-sampling. Running much longer simulations (on the *μ*s or ms order) would likely significantly influence the results due to conformational changes (see^72^ who showed that several properties extracted from MD simulations are stable over tens to hundreds of nanoseconds and that the microsecond timescale has to be reached to observe substantial changes). It may then be more pertinent to cluster the obtained conformational ensemble and run the analysis on each cluster.

Our approach is fully automated and can be applied to any pair of mutations, or triplets, not just point-wise mutations, for the analysis of combined mutational effects that might be deleterious but also compensatory (for the re-establishment of the function). It opens new avenues for developing efficient strategies to describe the mutational landscape of a protein from a structural perspective in a computationally tractable way. In principle, it is not limited to MD trajectories and can be applied to any conformational ensemble. Nevertheless, further investigations will be needed to determine whether all-atom MD can be replaced by more coarse-grained approaches without losing pertinent information. Even with MD simulations, this computational approach remains less costly than deep mutational scanning experiments. Our results and the increasing availability of validation data, from deep mutational scanning experiments or high-throughput exome sequencing, is very encouraging and let envisage large-scale applications of our approach.

## Materials and Methods

### Infostery analysis

All aspects of our infostery analysis were implemented in a fully automated tool, COMMA2 (www.lcqb.upmc.fr/COMMA2). COMMA2 is a new version of COMMA (COMmunication MApping), a method to describe the dynamical architecture of proteins and protein complexes^36^.

COMMA extracts residue-based properties from MD conformational ensembles and integrates them in a graph theoretic framework, where it identifies *dynamical units* (or communication blocks), *i*.*e*. groups of residues or protein regions that mediate information transmission across the protein structure^36^. These units are defined either based on *communication pathways* or on *independent cliques*.

*Communication pathways* are chains of residues complying with the following requirement: (*i*) two adjacent residues in the pathway are not adjacent in the sequence and (*ii*) form stable non-covalent interactions (hydrogen-bonds or hydrophobic contacts), (*iii*) any two residues in the pathway, adjacent or not, communicate efficiently. *Communication efficiency or propensity* is expressed as^36^:

1 $$CP(i,j)=\langle {({d}_{ij}-{\bar{d}}_{ij})}^{2}\rangle $$

where *d*_*ij*_ is the distance between the C*α* atoms of residues *i* and *j* and ${\bar{d}}_{ij}$ is the mean value computed over the set of conformations. Two residues *i* and *j* are considered to communicate efficiently if *CP*(*i*, *j*) is below a *communication propensity threshold*, CP_*cut*_. The strategy employed to set the value of *CP*_*cut*_ is detailed in^36^. Intuitively, neighbouring residues in the sequence forming well-defined secondary structures are expected to communicate efficiently with each other. First, we evaluate the proportion *p*_*ss*_ of residues that are in an *α*-helix, a *β*-sheet or a turn in more than half of the conformations. Then for every residue *i*, we compute a *modified communication propensity MCP*(*i*) as:

2 $$MCP(i)=\frac{1}{8}\,\sum _{\begin{array}{c}j=i-4\\ j\ne i;1\le j\le N\end{array}}^{i+4}\,CP(i,j)$$

where *N* is the total number of residues. *CP*_*cut*_ is chosen such that the proportion *p*_*ss*_ of *MCP* values are lower than *CP*_*cut*_.

As CP only accounts for residues’ relative displacements, two rigid residues will be considered as communicating efficiently, no matter where they are in the protein. But they will be linked by a pathway only if there exists a chain of residues linking them via pairwise stable interactions and communicating efficiently with them. Two residues adjacent in a pathway are said to be in direct communication. Two residues linked by a pathway but not adjacent are said to be in indirect communication. To avoid pathway redundancy, subpaths included in a longer pathway are discarded.

*Independent cliques* are clusters of residues where any two residues: (*i*) are close to each other in 3D space (minimum inter-atomic distance smaller than 3.7 Å) and (*ii*) display high concerted atomic fluctuations. Each *independent clique* is comprised of residues that are highly flexible relative to the rest of the protein, and that fluctuate in a concerted way (high correlations between them) independently from the rest of the protein (low correlations with the other residues). Precise definitions of the measures and algorithms used to construct independent cliques are given in^36^.

*Communication pathways* and *independent cliques* are used to construct a coloured graph PCN(*N*, *E*) defined by nodes *N* that correspond to the residues of the protein and edges *E* that connect residues adjacent in a pathway or belonging to the same clique. COMMA extracts connected components from the graph by using depth-first search (DFS) to identify the protein *dynamical units*. These units are referred to as “communication blocks” in^36^.

COMMA2 implements several new functionalities compared to COMMA: (*i*) computation of a persistency score for each residue, (*ii*) automated detection of residues bridging pathway-based and clique-based *dynamical units*, (*iii*) automated generation of dot plots displaying all detected *direct* and *indirect communications*, (*iv*) automated detection of *isolated direct communications*. The algorithms associated to those functionalities are explained in the following.

#### Residue persistency scores

*Communication pathways* and *independent cliques* are defined by setting several parameters, namely the *communication propensity threshold*, *CP*_*cut*_, and the *local feature analysis correlation threshold*, $Cor{r}_{cut}^{LFA}$^36^. Default values are attributed to these parameters, depending on the studied system^36^. COMMA2 implements a procedure that systematically considers ranges of values for *CP*_*cut*_ and $Cor{r}_{cut}^{LFA}$ to detect *dynamical units*. It then computes the propensity of each residue to be detected in a *dynamical unit*, as the number of times the residue was included in a unit over the total number of parameters values considered. The default procedure is to vary the thresholds from their default value up to the value where all residues of the protein are in the same unit, by increments of 5% of the distributions used to define them (*MCP* and *Corr*^*LFA*^, see^36^). The procedure is customizable by the user. *Persistency scores* enable assessing the robustness of the results with respect to parameter variations.

#### Algorithm to detect residues bridging pathway-based and clique-based dynamical units

Residues bridging pathway-based and clique-based *dynamical units* are defined as residues that are detected in a pathway-based unit and also in a clique-based unit with *persistency scores* greater than 80% (default value). This cutoff value is customizable by the user.

#### Dot plot displaying all direct and indirect communications

Given a value of *CP*_*cut*_, COMMA2 creates a dot plot that displays all the *direct* and *indirect communications* detected in the studied system (see Fig. 5 for an example). The black and grey dots correspond to pairs of residues that are in *direct communication* (adjacent in a path). If the two residues are separated by less than 4 residues in the primary sequence, they are in grey, otherwise, they are in black. Colored dots correspond to pairs of residues that are in *indirect communication* (in the same path, but not adjacent). Each color stands for a dynamical unit.

#### Algorithm for picking up isolated direct communications

*Isolated direct communications* are detected between pairs of residues that communicate faster than their context (residues around them). To detect them, the *communication propensity threshold*, *CP*_*cut*_, is varied from its default value up to the value where all residues of the protein are in the same unit (typically 80% of the *MCP* distribution, see^36^ for the definition of *MCP*), by increments of 5%. This level of resolution proved sufficient to record essentially all significant changes in the dot plot of *direct* and *indirect communications*. The algorithm extracts groups of isolated black dots (from 1 to 5) from each dot plot. For this, we define a motif as a group of points in which each point is adjacent to at least one other point from the group. A black dot will be considered as isolated either if it is not part of any motif (it is isolated *stricto sensu*), or if the motif to which it belongs contains: (*i*) no more than 5 black dots, (*ii*) no grey dot and (*iii*) no more than 4 colored dots. These values were empirically chosen and proved suitable for our systems. Other systems may require adjustments.

### Molecular dynamics simulations

#### Set up of the systems

The 3D coordinates of PSD95^*pdz*3^ in complex with its cognate ligand, a C-terminal peptide derived from CRIPT, were retrieved from the Protein Data Bank^73^ (PDB code: 1BE9, residues 302 to 430, 1.82 Å resolution^74^). The CRIPT peptide (sequence: TKNYKQTSV, residues -8 to 0) is truncated in the PDB structure (sequence: KQTSV) and the missing residues and side chains were modeled using MODELLER 9v7^75^. 175 mutations (Supplementary Table S1) were applied by *in silico* substitutions using RosettaBackrub^76^. PSD95^*pdz*3^ domain contains 2 histidines, whose protonation states were determined so as to locally optimize the hydrogen-bond network: (*i*) a hydrogen was assigned to the $\epsilon$-nitrogen of H317 and (*ii*) a hydrogen was assigned to the *δ*-nitrogen of H372.

The 3D coordinates of TEM-1 were retrieved from the PDB entry 1XPB^77^ (chain A, residues 26 to 290, 1.9 Å resolution). TEM-1 contains 6 histidines, whose protonation states were determined so as to locally optimize the hydrogen-bond network: (i) a hydrogen was assigned to the $\epsilon$-nitrogen of H26, H112, H158 and H289 and (ii) a hydrogen was assigned to the *δ*-nitrogen of H96 and H153.

The 3D coordinates of the GH-GHR complex were retrieved from the PDB entry 1HWG^78^ (191 residues for GH, chain A, 237 residues for each receptor, chains B and C, 2.9 Å resolution). Missing residues, namely 148–153 and 191 of GH and 54–62 of GHR were modelled with MODELLER 9v7^75^. All crystallographic water molecules and other non-protein molecules were removed. The 8 disulphide bonds present in the complex, namely (C53, C165) and (C182, C189) in GH, (C38, C48), (C83, C94) and (C108, C122) in GHR1 and GHR2, were kept. The environment of the histidines was manually checked and they were consequently protonated with a hydrogen at the $\epsilon$ nitrogen.

#### Preparation

All crystallographic water molecules and other non-protein molecules were removed. All systems were prepared with the LEAP module of AMBER 12^79^, using the ff12SB forcefield parameter set: (*i*) hydrogen atoms were added, (*ii*) the solute was hydrated with a cuboid box of explicit TIP3P water molecules with a buffering distance up to 10 Å, (*iii*) Na^+^ and Cl^−^ counter-ions were added to reproduce physiological salt concentration (150 mM solution of potassium chloride).

#### Minimization, heating and equilibration

The systems were minimized, thermalized and equilibrated using the SANDER module of AMBER 12. The following minimization procedure was applied: (*i*) 10,000 steps of minimization of the water molecules keeping protein atoms fixed, (*ii*) 10,000 steps of minimization keeping only protein backbone fixed to allow protein side chains to relax, (*iii*) 10,000 steps of minimization without any constraint on the system. Heating of the system to the target temperature of 310 K was performed at constant volume using the Berendsen thermostat^80^ and while restraining the solute *C*_*α*_ atoms with a force constant of 10 *kcal*/*mol*/*Å*^2^. Thereafter, the system was equilibrated for 100 *ps* at constant volume (NVT) and for further 100 *ps* using a Langevin piston (NPT)^81^ to maintain the pressure. Finally the restraints were removed and the system was equilibrated for a final 100 *ps* run.

#### Production of the trajectories

The simulations were realized in the NPT ensemble using the PMEMD module of AMBER 12. The time step was set to 2.0 fs. The temperature was kept at 310 K and pressure at 1 bar using the Langevin piston coupling algorithm. The SHAKE algorithm was used to freeze bonds involving hydrogen atoms, allowing for an integration time step of 2.0 fs. The Particle Mesh Ewald (PME) method^82^ was employed to treat long-range electrostatics. The coordinates of the system were written every ps. For each system of PSD95^*pdz*3^-CRIPT peptide complex, 5 replicates of 20 ns were performed, starting with different initial velocities. For TEM-1, 2 replicates of 50 ns were produced. For GH-GHR complex, 2 replicates of 100 ns were produced. See Supplementary Table S4 for simulation details.

#### Stability of the trajectories

Standard analyses of the MD trajectories were performed with the *ptraj* module of AMBER 12. The all-atom root mean square deviation (RMSD) from the initial frame, were recorded along each replicate. Based on the RMSD profiles, we performed the subsequent analyses over the last 15 ns of each replicate for PSD95^*pdz*3^-CRIPT peptide complex, the last 45 ns for TEM-1 and the last 70 ns for GH-GHR complex. The by-residue root mean square fluctuations (RMSF) was computed with respect to the average conformation. The secondary structures were assigned with DSSP^83^. All the studied systems proved to remain stable in the MD trajectories.

#### Residue burial

The degree of burial of protein residues was estimated by the circular variance. Circular variance (CV) is a measure of the vectorial distribution of a set of neighboring points around a fixed point in 3D space^84^. For a given residue, CV reflects the density of protein around it. CV has the advantage of changing more smoothly than surface accessibility in passing from the surface to the interior of the protein^85^, making it less sensitive to small conformational changes. CV can be applied equally well to atomic or coarse-grain representations^84^. The CV value of an atom *i* is computed as:

3 $$CV(i)=1-\frac{1}{{n}_{i}}|\sum _{j\ne i,{r}_{i}\le {r}_{c}}\frac{{\overrightarrow{r}}_{ij}}{\parallel {\overrightarrow{r}}_{ij}\parallel }|$$

where *n*_*i*_ is the number of atoms distant by less than *r*_*c*_Å from atom *i*. The CV value of a residue *j* is then computed as the average of the atomic CVs, over all the atoms of *j*. A low CV value indicates for a residue that it is located in a protruding region of the protein surface. CV values are scaled between 0 (most protruding residue of the protein) and 1 (least protruding residue of the protein) for the calculation of residue scores. The cutoff distance *r*_*c*_ directly influences the resolution of the protein surface. Here we chose *r*_*c*_ = 20 *Å* and we consider a residue to be buried within the protein when its CV value is higher or equal to *CV*_*cut*_ = 0.6. These parameters were calibrated by comparing CV values and solvent accessible surface areas. The threshold *CV*_*cut*_ roughly corresponds to 20–25% solvent accessibility.

#### Algorithm for defining convex hull from the network of communication pathways

All pathways longer than 3 residues were considered. The edges between pairs of residues where at least one residue of the pair is not connected to any other residue were removed iteratively, until no such edge was present in the network. The remaining network was mapped onto the average MD conformation, for each system. The volume of the convex hull was computed using the 3D coordinates of C*α* atoms of the residues in this sub-network.

### Sequence analysis

Evolutionary conservation was computed using JET^65^. Starting from the query sequence, JET retrieves homologous sequences and sample them with a Gibbs-like approach^65^. *N* trees are constructed from *N* representative subsets of sequences. For each position in the query sequence, a tree trace is computed from each tree *T*: it corresponds to the level *n* in the tree *T* where the amino acid at this position appeared and remained conserved thereafter^65^. Tree traces are averaged over the *N* trees to get more statistically significant values. The final T_*JET*_ value of amino acid *a*_*j*_ at position *j* is obtained by accounting for *a*_*j*_’s environment^65^. T_*JET*_ values are scaled between 0 (least conserved residue of the protein) and 1 (most conserved residue of the protein). JET was applied to the sequences of PSD95^*pdz*3^, GH and TEM-1. Residues displaying T_*JET*_ values above 0.7 were considered as highly conserved.

Coevolved residues were detected in PSD95^*pdz*3^ by SCA^66^, DCA^67^ and MST^68^. They were respectively taken from^3^, from^6^ and from^68^.

### Experimental datasets

#### PSD95^*pdz*3^-CRIPT peptide complex

We used the matrix of 20 (amino acid types) × 83 (positions) experimental Δ*E* values reported in^3^ as our reference for defining beneficial, neutral and deleterious mutations. These values correspond to binding affinity changes between PSD95^*pdz*3^ and its cognate ligand, the CRIPT peptide, upon every possible single amino-acid substitution. They were indirectly estimated by measuring the frequencies of mutated alleles in a bacterial population where cells were classified based on their content of PSD95^*pdz*3^-CRIPT peptide complex (assessed by eGFP levels). The Δ*E* values range from −1.89 kcal/mol (highly deleterious) to 0.34 kcal/mol (beneficial). The values reported for the wild-type amino acids are not exactly zero but vary between −0.17 and 0.18 kcal/mol. This range gives an idea of the experimental noise contained in the data.

We considered the 20 highly sensitive positions identified in^3^: L323, G324, F325, I327, V328, G329, G330, I336, I338, I341, A347, L353, I359, V362, L367, H372, A375, A376, L379, I388. The 175 studied substitutions were classified as neutral (Δ*E* ≥ −0.2 kcal/mol), deleterious (Δ*E* < −0.2 kcal/mol) and highly deleterious (Δ*E* < −1.0 kcal/mol). Sequence analysis was used to define a restricted set of mutations. 1384 sequences homologous to that of PSD95^*pdz*3^ were retrieved by a PSI-BLAST^86^ search (3 iterations, e-value < 10^−5^) and aligned ClustalW^87^. The restricted set comprised 15 neutral mutations (Δ*E* ≥ −0.2 kcal/mol) found in more than 30 homologous sequences, and 41 highly deleterious mutations (Δ*E* < −1.0 kcal/mol) found in less than 10 sequences.

#### TEM-1

We used the experimental data reported in^4^. In this experiment, the protein fitness landscape was characterized by measuring the relative abundance of each possible TEM-1 mutation in a population of cells subjected to antibiotic treatment. The authors estimated the sensitivity of every position to mutations by computing a *k*$\ast$ value that reflects the number of mutations leading to inactivation of the protein^4^. They identified 8 positions as highly sensitive to mutations ($k\ast  < 2.5$): S70, K73, S130, D131, E166, D179, T181, K234, G251.

#### Growth hormone

As no experimental measurements of single-point mutation effects were available for GH, the set of putative sensitive positions was defined as have an evolutionary conservation value *T*_*JET*_ > 0.7 and less than 5 alleles bearing missense mutations reported in the Exome Aggregation Consortium (ExAC) database^60^. ExAC contains exome sequencing data from more than 60 000 unrelated individuals. We identified 30 positions: A17, A24, F31, C53, F54, S55, I58, L75, L76, S79, L82, I85, W86, P89, V90, L93, L114, L117, G120, L124, L162, C165, F166, K168, D169, K172, E174, E175, L177, V180.

### Other tools

PyMOL^88^ was used for visualization and the analyses were performed using the R software^89^. ENCoM^63^ was used according to the protocol reported in^64^ and described in details in^90^. This protocol performs *in silico* mutation using MODELLER^75^, infer protein motions using normal mode analysis, and predict mutational outcome by computing a ΔΔ*G* value as a linear combination of ENCoM prediction and FOLDX^91^ folding energy change. Mutations with ΔΔ*G* > 0 are predicted as deleterious. STRESS web server^49^ was used with default parameters. Given an input PDB structure, it determines a set of surface-critical residues and a set of interior-critical residues. Only the residues identified as interior-critical were considered.

## Electronic supplementary material

Supplementary file
