# Supplementary material for: “Infostery” analysis of short molecular dynamics simulations identifies highly sensitive residues and predicts deleterious mutations
Source: Sci Rep. 2018 Oct 31;8:16126. doi: 10.1038/s41598-018-34508-2 (PMC6208415; doi:10.1038/s41598-018-34508-2)
Supplement: Supplementary file 1 — Supplementary file [file 41598_2018_34508_MOESM1_ESM.pdf]

"Infostery" analysis of short molecular dynamics simulations identifies highly sensitive residues and predicts deleterious mutations

Yasaman Karami[1,2], Tristan Bitard-Feildel[1,2], Elodie Laine[1,*], and Alessandra Carbone[1,3,*]

[1]Sorbonne Université, CNRS, IBPS, Laboratoire de Biologie Computationnelle et Quantitative (LCQB), 75005 Paris, France.
[2]Sorbonne Université, Institut des Sciences du Calcul et de des Données
[3]Institut Universitaire de France
[*]elodie.laine@upmc.fr, alessandra.carbone@lip6.fr

ABSTRACT

Characterizing a protein mutational landscape is a very challenging problem in Biology. Many disease-associated mutations do not seem to produce any effect on the global shape nor motions of the protein. Here, we use relatively short all-atom biomolecular simulations to predict mutational outcomes and we quantitatively assess the predictions on several hundreds of mutants. We perform simulations of the wild type and 175 mutants of PSD95's third PDZ domain in complex with its cognate ligand. By recording residue displacements correlations and interactions, we identify "communication pathways" and quantify them to predict the severity of the mutations. Moreover, we show that by exploiting simulations of the wild type, one can detect 80% of the positions highly sensitive to mutations with a precision of 89%. Importantly, our analysis describes the role of these positions in the inter-residue communication and dynamical architecture of the complex. We assess our approach on three different systems using data from deep mutational scanning experiments and high-throughput exome sequencing. We refer to our analysis as "infostery", from "info" - information - and "steric" - arrangement of residues in space. We provide a fully automated tool, COMMA2 (www.lcqb.upmc.fr/COMMA2), that can be used to guide medicinal research by selecting important positions/mutations.

Position	Wild-type aa	Applied substitutions
311	P	W
323	L	A, D, F, G, H, I, K, M, N, Q, R, S, T, V, W, Y
324	G	A, C, D, E, F, H, I, K, L, N, P, Q, R, S, T, V, W, Y
325	F	A, C, D, E, G, H, I, L, M, N, P, Q, S, T, V, W
329	G	A, C, D, E, F, H, I, K, L, M, N, P, Q, R, S, T, V, W
330	G	A, C, D, F, H, I, K, L, M, N, Q, R, S, T, V, Y
336	I	C, D, E, F, G, H, L, N, P, Q, R, S, T, V, W
338	I	A, C, D, E, F, G, H, K, L, M, N, P, Q, R, S, T, V, W, Y
341	I	A, C, D, E, F, G, H, K, L, M, N, P, Q, R, S, T, V, W, Y
347	A	C, D, E, F, G, H, I, K, L, M, N, P, Q, R, S, T, V, W, Y
372	H	A, C, D, E, F, G, I, K, L, M, N, P, Q, R, S, T
366	D	A
371	S	A

Table S1. List of studied mutations.

Protein	Experimental ΔE (in kcal/mol)[1]	Gain of pathways (> 3 residue long)	Convex hull	
			area (in Å^2)	volume (in Å^3)
WT	[-0.17 ; 0.18]	-	2079	5248
MU^{P311W}	0.31	63	1871	4779
MU^{S371A}	0.02	128	2053	5060
MU^{F325A}	-0.03	-39	2032	5094
MU^{I341A}	-0.64	455	1863	4452
MU^{H372A}	-1.34	566	2211	5959
MU^{G329A}	-1.36	587	2121	6906
MU^{A347F}	-1.42	819	2087	6792

Table S2. Seven chosen mutations. The experimental measurements are given in the first column. In the second column are reported the number of communication pathways (> 3 residue long) gained compared to the wild-type complex. In the third and fourth columns, the area and volume of the convex hull defined from the network of communication pathways are given. The convex hull was defined by mapping the network of all pathways (> 3 residue long) onto the averaged MD conformation and removing the isolated branches.

Protein	#(residues)	#(distant pairs)	quantile	#(indirect com.)	#(direct com.)
PSD95^{pdz3}	83	6 216	65%	203 (3.27%)	70 (1.13%)
			70%	274 (4.41%)	76 (1.22%)
			75%	437 (7.03%)	84 (1.35%)
			80%	896 (14.41%)	109 (1.75%)
TEM-1	263	33 670	65%	144 (0.43%)	96 (0.29%)
			70%	432 (1.28%)	141 (0.42%)
			75%	471 (1.40%)	149 (0.44%)
			80%	806 (2.39%)	171 (0.51%)
GH	191	17 578	65%	330 (1.09%)	14 (0.08%)
			70%	511 (2.91%)	21 (0.12%)
			75%	745 (4.24%)	35 (0.20%)
			80%	1 480 (8.42%)	73 (0.42%)

Table S3. Indirect and direct communication statistics. For each protein, the number of residues, the number of residue pairs far away in the sequence ($>$ 3 residues apart), the quantiles of the communication propensity distribution used to set the communication propensity thresholds (see *Materials and Methods*, and the corresponding number of detected indirect and direct communications (percentage of residue pairs in parenthesis) are given.

Protein or protein complex	PDB code	#(residues)	#(systems)	#(replicates)	Time (ns)	Total time (ns)
PSD95^{pdz3}-CRIPT peptide	1BE9	124	176	5	20	17 600
PSD95^{pdz3}	1BE9	115	2	2	20	80
TEM-1	1XPB	263	1	2	50	100
GH-GHR	1HWG	569	1	2	100	200

Table S4. Details of the simulations.

Residue	Connected component	Tolerance to mutations[2]	Evolutionary conservation	Catalytic cleft
K73	black	2.11	0.97	✓
V74	black	8.76	0.56	
A126	black	8.08	0.62	
I127	black	6.79	0.54	
T128	black	15.98	0.63	
D131	black	1.14	0.98	✓
N132	black	4.78	0.90	✓
N136	black	9.00	0.92	
E166	black	2.08	0.94	✓
W210	black	9.32	0.83	
M211	black	5.07	0.66	
D214	black	13.3	0.73	
A232	black	7.17	0.74	
K234	black	2.24	0.84	✓
S235	black	5.23	0.75	✓
F66	blue	4.6	0.93	
P67	blue	12.61	0.71	
M69	blue	12.97	0.69	
S70	blue	1.15	0.94	✓
T71	blue	6.09	0.82	
T180	blue	5.48	0.90	
T181	blue	2.83	0.77	
G236	blue	4.63	1	✓
A237	blue	7.86	0.57	
S243	blue	9.64	0.59	
R244	blue	8.26	0.66	
Y264	blue	5.58	0.73	
L169	green	10.68	0.81	
D179	green	2.09	0.90	
I47	cyan	11.49	0.55	
P183	cyan	5.96	0.83	
A187	cyan	6.16	0.61	
N170	yellow	12.40	0.86	✓
G238	yellow	5.97	0.69	

Table S5. Detection of residues forming isolated communication bridges in TEM-1. For each detected residue, we report the connected component to which it belongs (the color code is the same as in **Supplementary Figure S9**), its tolerance to mutations ($k*$ defined in[2]), its conservation degree (calculated by JET, see *Materials and Methods*) and whether it belongs to the catalytic cleft.

Strategy	Tolerance $k*$[2]	Sens	PPV	Spe	Acc
Infostery:	$\leq$2.5	75	18	89	89
isolated direct	$\leq$5	48	29	90	87
communications	$\leq$10	33	79	96	76

Table S6. Performance of infostery to detect sensitive positions in TEM-1. The performance values, sensitivity (*Sens*), precision or positive predictive value (*PPV*), specificity (*Spe*) and accuracy (*Acc*), are given in percentages. Only isolated direct communications were detected.

Positions	infostery-based strategy
A17	3
A24	3
F31	3
C53*	3
F54	-
S55	3
I58	-
L75	3
L76	-
S79	3
L82	3
I85	3
W86	3
P89	-
V90	3
L93	3
L114	3
L117	3
G120	-
L124	-
L162	3
C165*	3
F166	-
K168	-
D169	3
K172	
E174	3
T175*	2
L177	3
V180	3

Table S7. List of putative sensitive positions in growth hormone. The positions were detected by sequence analysis across species and across patients (see *Materials ad Methods*). Those highlighted by a star were identified experimentally as crucial for the interaction between growth hormone and its receptor[3,4]. The infostery-based strategies are: (1) belonging to both a path-based unit and a clique-based unit, (2) direct communication with the partner, (3) isolated direct communication within the protein. The numbering corresponds to the PDB entry 1HWG[5].

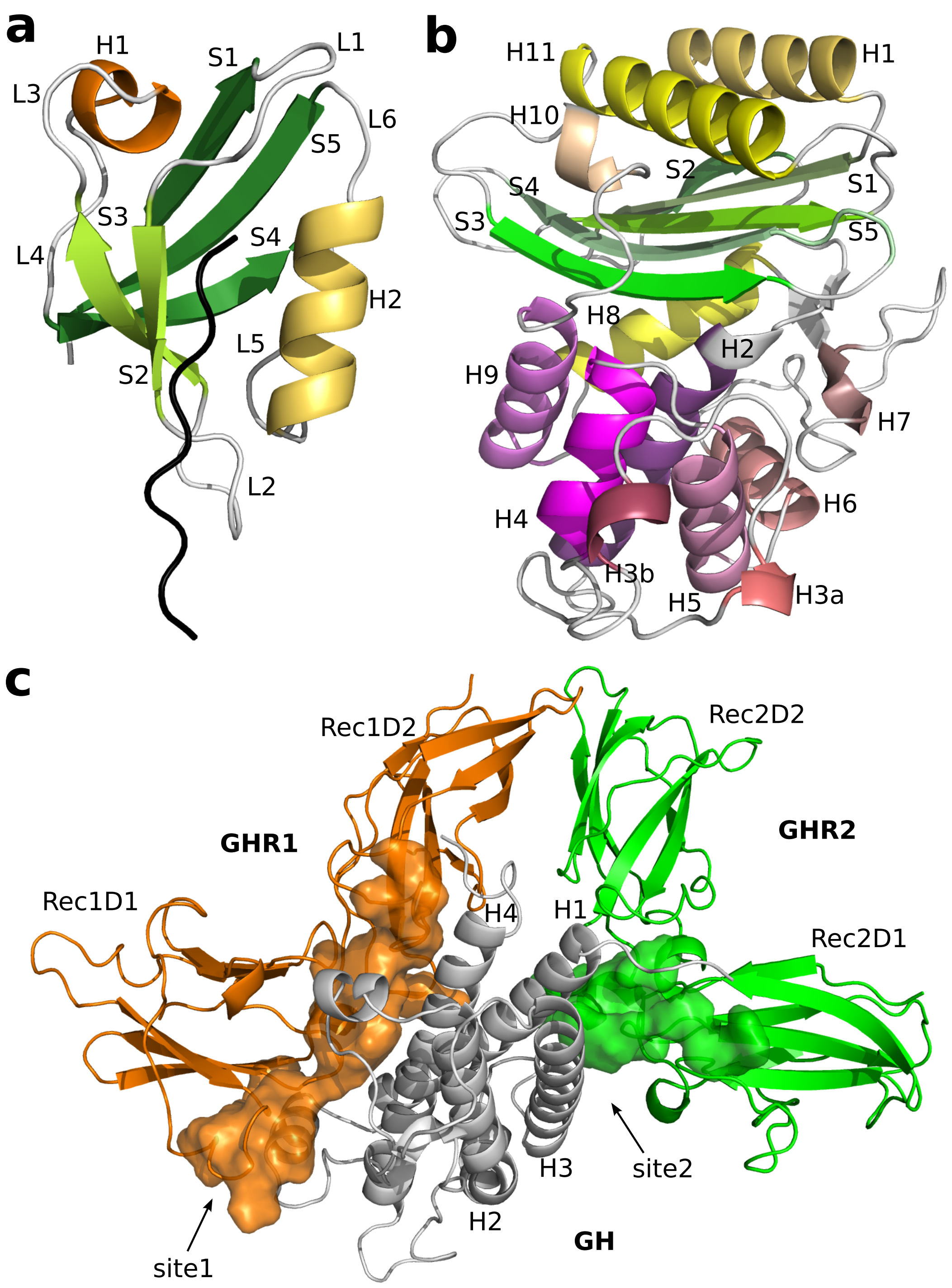

Figure S1. Tertiary structures of the three protein and protein complexes under study. **(a)** PSD95^{pdz3}-CRIPT peptide complex (PDB code: 1BE9[6]). **(b)** TEM-1 (PDB code: 1XPB[7]). **(c)** GH-GHR complex (PDB code:1HWG[5]).

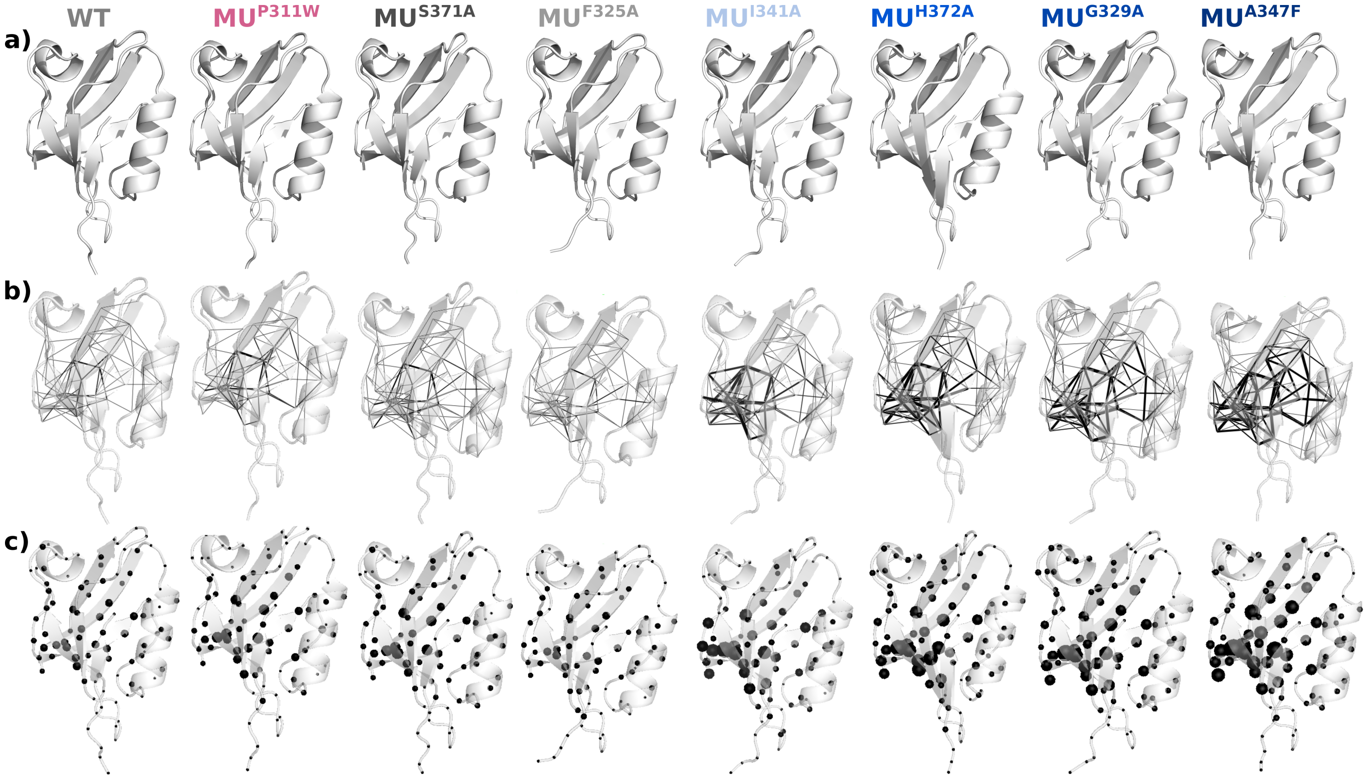

Figure S2. Infostery analysis for PSD95^{pdz3}-CRIPT peptide complex. **(a)** Conformations averaged over 5 replicates of 15-ns MD simulations of the wild-type complex (WT) and 7 mutants. **(b)** Communication pathways (> 3 residues) mapped onto the averaged conformation and displayed as black lines. The thickness of each segment is proportional to the number of pathways linking the two residues. **(c)** The residues crossed by at least one communication pathway (> 3 residues) are displayed as black spheres, centered on their C-α atoms. The size of each sphere is proportional to the number of pathways crossing the residue. The labels are colored according to the experimentally assessed effects of the mutations (beneficial in pink, neutral in grey, deleterious in blue).

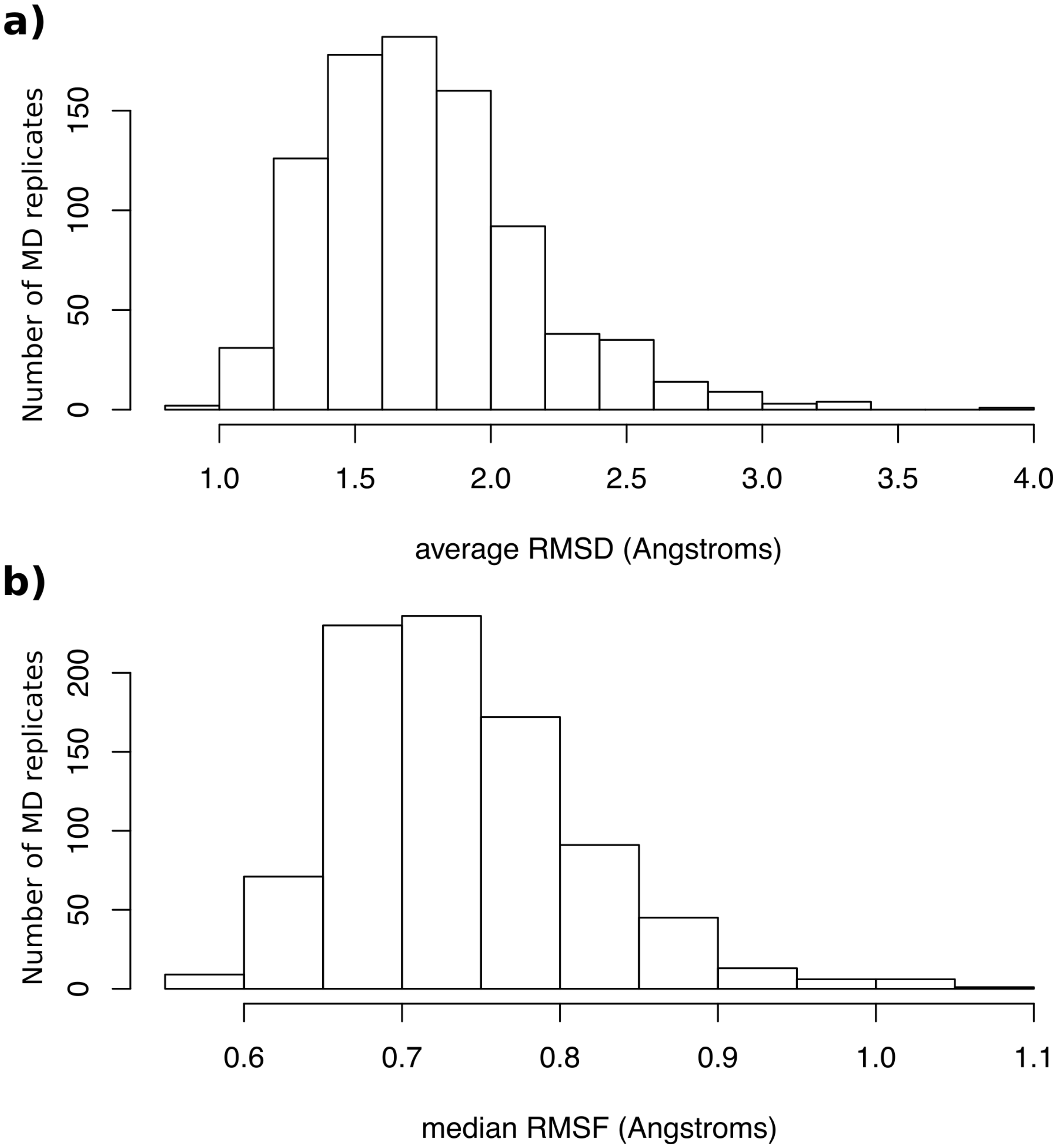

Figure S3. Root mean square deviations and fluctuations for PSD95^{pdz3}-CRIPT peptide complex. Distributions of the averaged RMSD values (**a**) and the median RMSF values (**b**), computed over each of the 5 replicates of MD simulation for the wild-type complex and its 175 mutants (880 points in total). The RMSD was computed on all atoms, with respect to the initial frame. The RMSF was computed on all atoms and averaged by residue, considering the last 15 ns and with respect to the average conformation. Each RMSF value in the distribution is the median computed over the protein (residues 311 to 393) for one MD simulation.

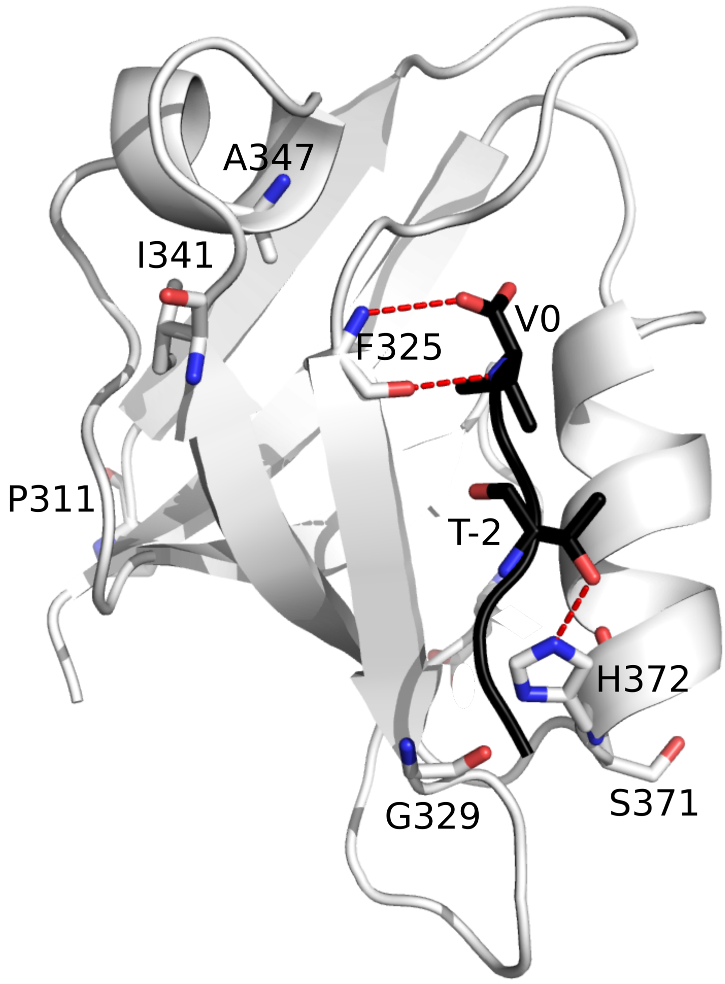

Figure S4. Localization of the studied mutations in the PSD95^{pdz3}-CRIPT peptide complex. The protein PSD95^{PDZ3} and the ligand (PDB code: 1BE9) are displayed as cartoons colored in white and black respectively. The residues whose mutations were studied and residues from the ligand with which they interact are shown as sticks. Hydrogen-bonds are indicated as dashed red lines.

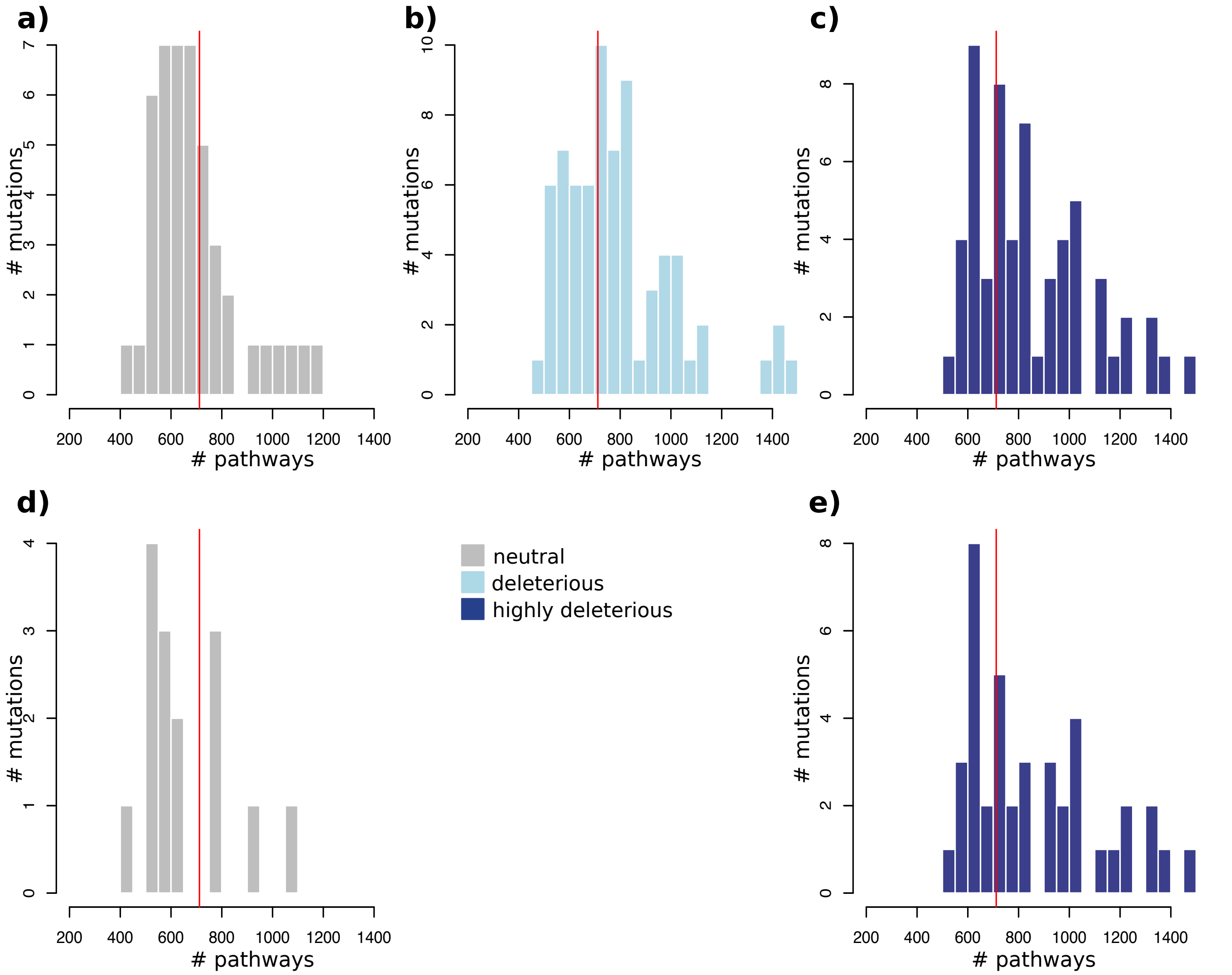

Figure S5. Distributions of the number of pathways. **(a)** 45 neutral mutations ($\Delta E \geq -0.2$ kcal/mol). **(b)** 71 deleterious mutations ($\Delta E < -0.2$ kcal/mol). **(c)** 59 highly deleterious mutations ($\Delta E < -1.0$ kcal/mol). **(d)** 15 neutral mutations that appear in more than 30 (over 1384, 2%) homologous sequences. **(e)** 41 highly deleterious mutations that appearing in less than 10 (over 1384, 0.7%) homologous sequences. In total, 175 mutations are considered, and only the pathways longer than 3 residues are retained.

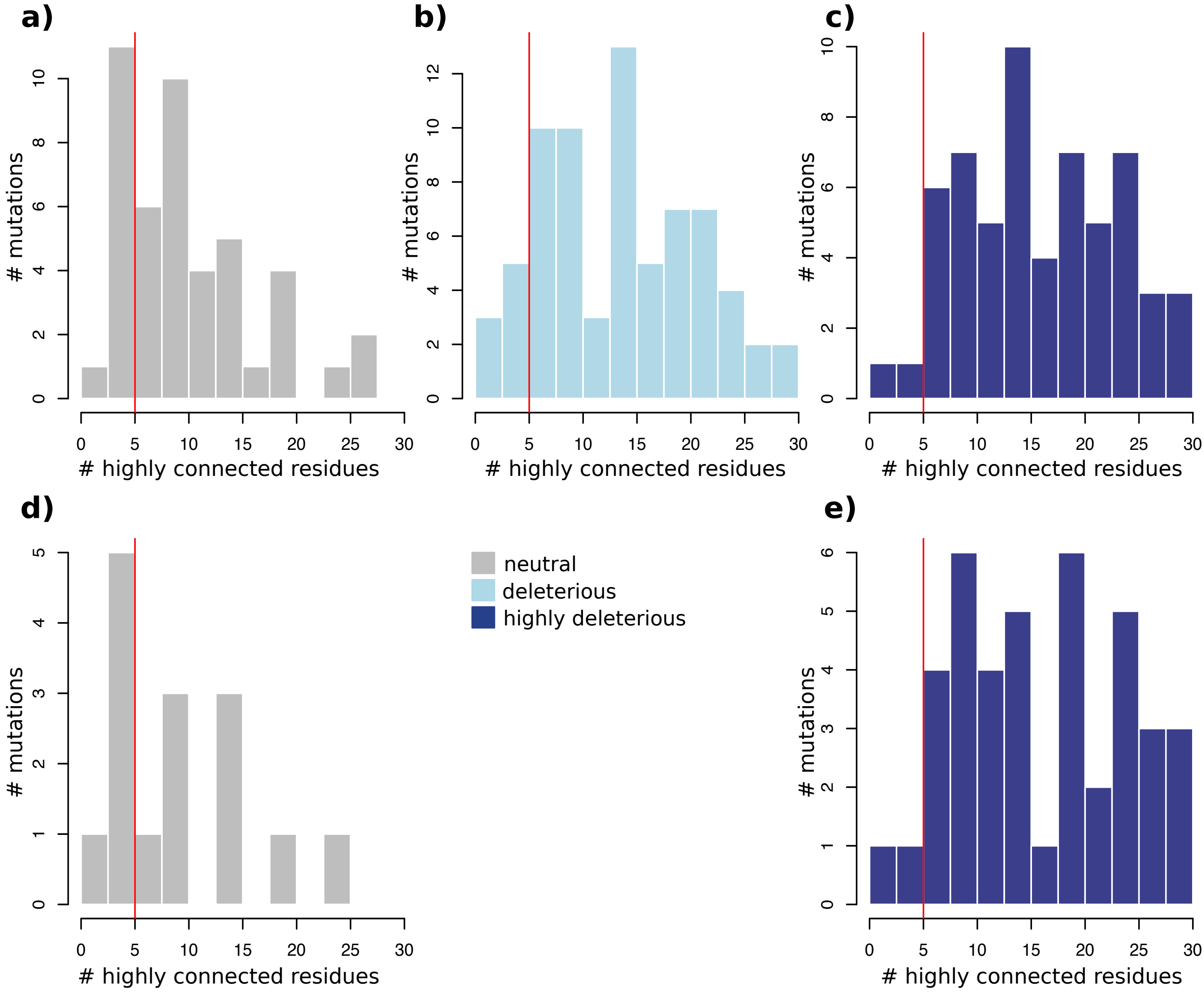

Figure S6. Distributions of the number of highly connected residues. **(a)** 45 neutral mutations ($\Delta E \geq -0.2$ kcal/mol). **(b)** 71 deleterious mutations ($\Delta E < -0.2$ kcal/mol). **(c)** 59 highly deleterious mutations ($\Delta E < -1.0$ kcal/mol). **(d)** 15 neutral mutations that appear in more than 30 (over 1384, 2%) homologous sequences. **(e)** 41 highly deleterious mutations that appearing in less than 10 (over 1384, 0.7%) homologous sequences. In total, 175 mutations are considered, and highly connected residues are those crossed by > 70 pathways. The red lines indicate the value computed for the wild type.

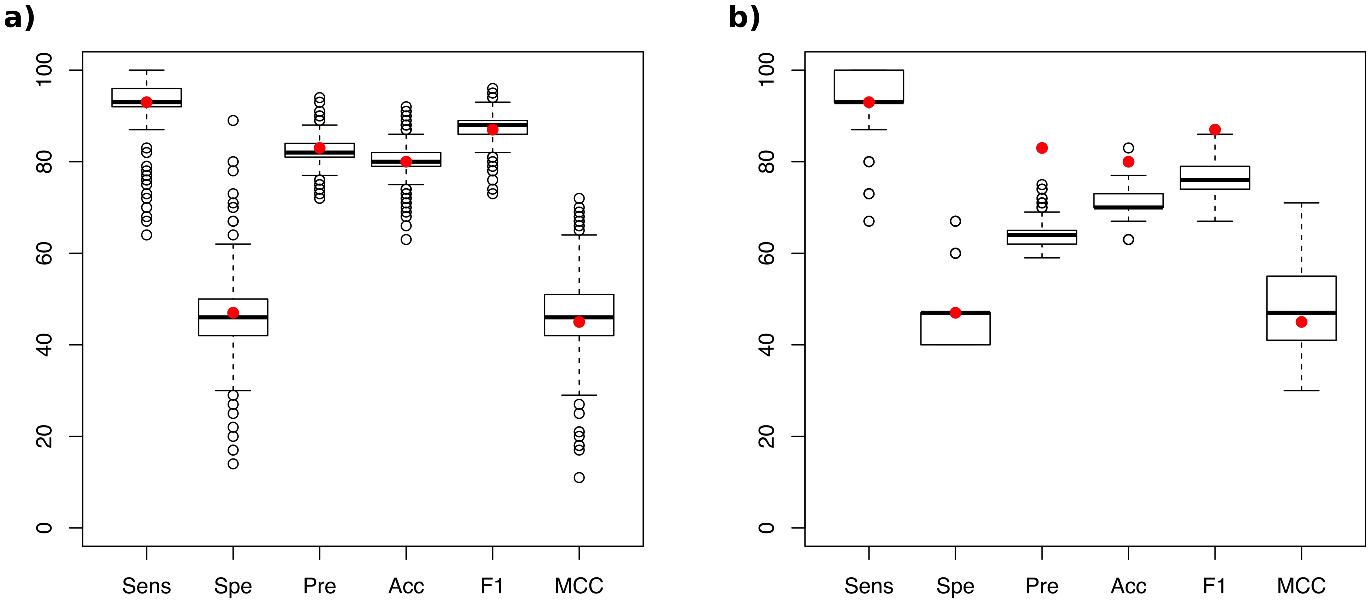

Figure S7. Robustness of the predictive performance of our infostery approach. (a) Distributions of performance values (sensitivity, specificity, precision, accuracy, F1-score and Matthews correlation coefficient) over 500 random subsets of mutations of varied lengths, extracted from the original set of 41 highly deleterious and 15 neutral mutations (see Table I). The average ratio between numbers of highly deleterious and neutral mutations is 2.79 (for comparison, 41/15=2.73). **(b)** Distributions of performance values over 500 random balanced subsets of mutations (15 highly deleterious and 15 neutral). The red dots indicate the performance obtained on the original set of 41 highly deleterious and 15 neutral mutations (see Table I, coef=1.2).

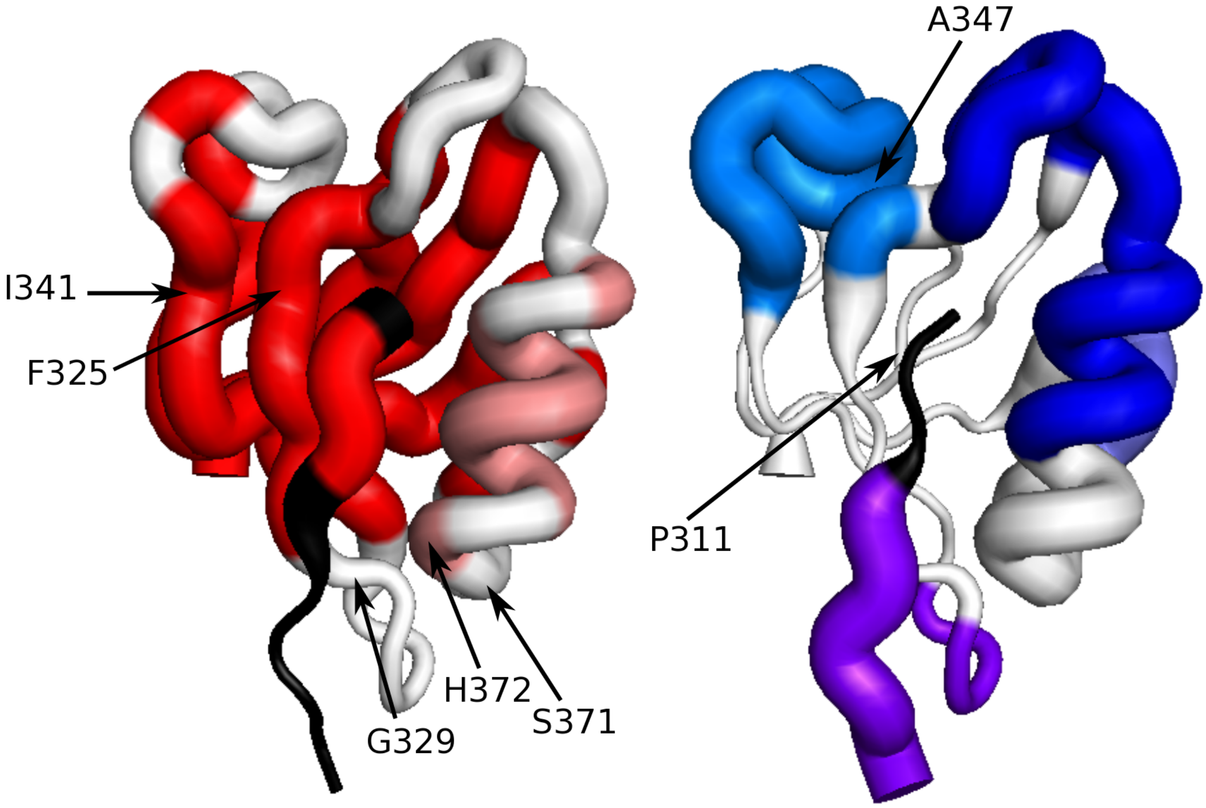

Figure S8. Dynamical units in wild-type PSD95^{pdz3}-CRIPT peptide complex. On the left: 2 pathway-based units colored in red and pink. On the right: 4 clique-based units colored in different blue tones. The protein is in white and the ligand in black. The size of the sausage reflects the propensity of each residue to be detected in a CB (see *Materials and Methods*).

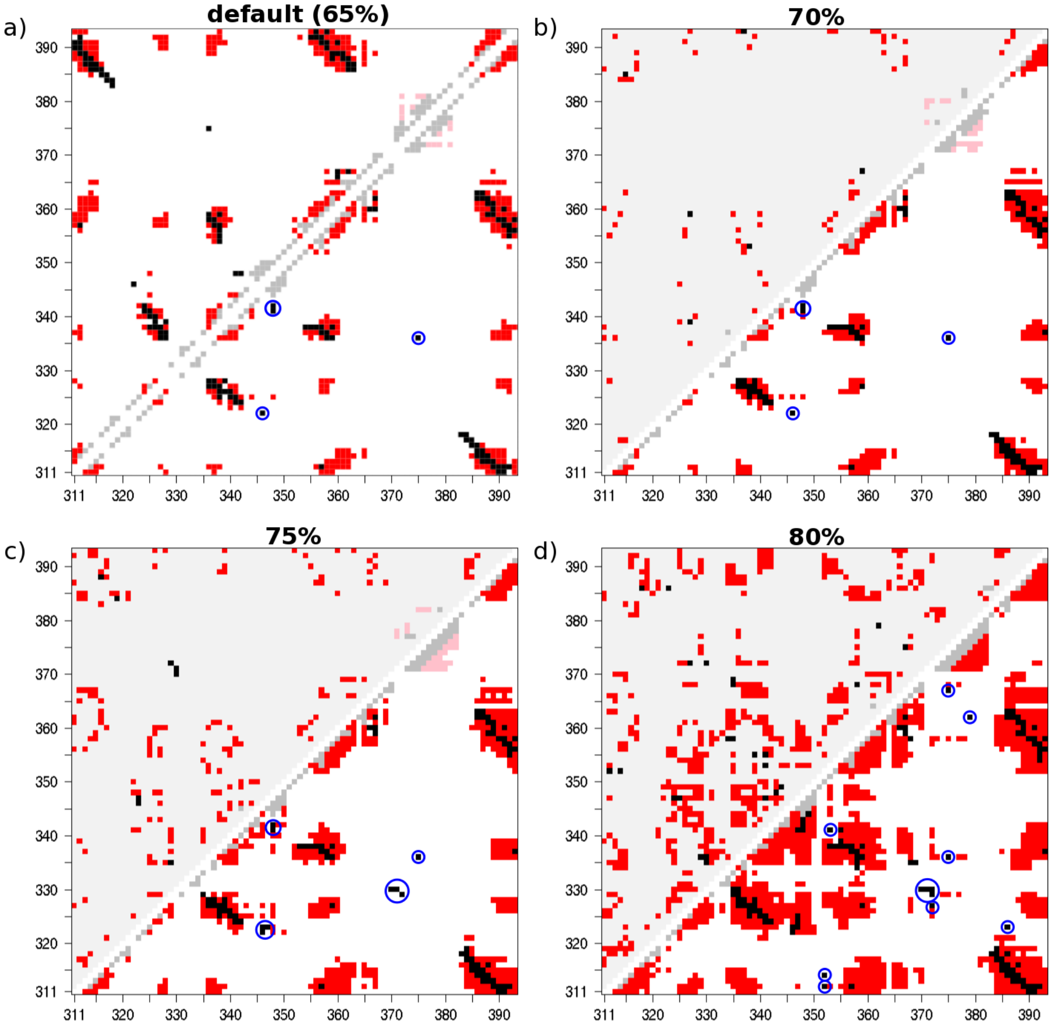

Figure S9. Dotplots representing direct and indirect communications between PDZ residues. In the lower right triangles are displayed the direct and indirect communications obtained by setting the communication propensity threshold at the values corresponding to 65% (a), 70% (b), 75% (c) and 80% (d) quantile of the distribution. In the upper left triangles are displayed only the communications that are added compared to the immediately preceding threshold value, except for the first dotplot (a), which is symmetrical. The legend is the same as in **Fig. 4**.

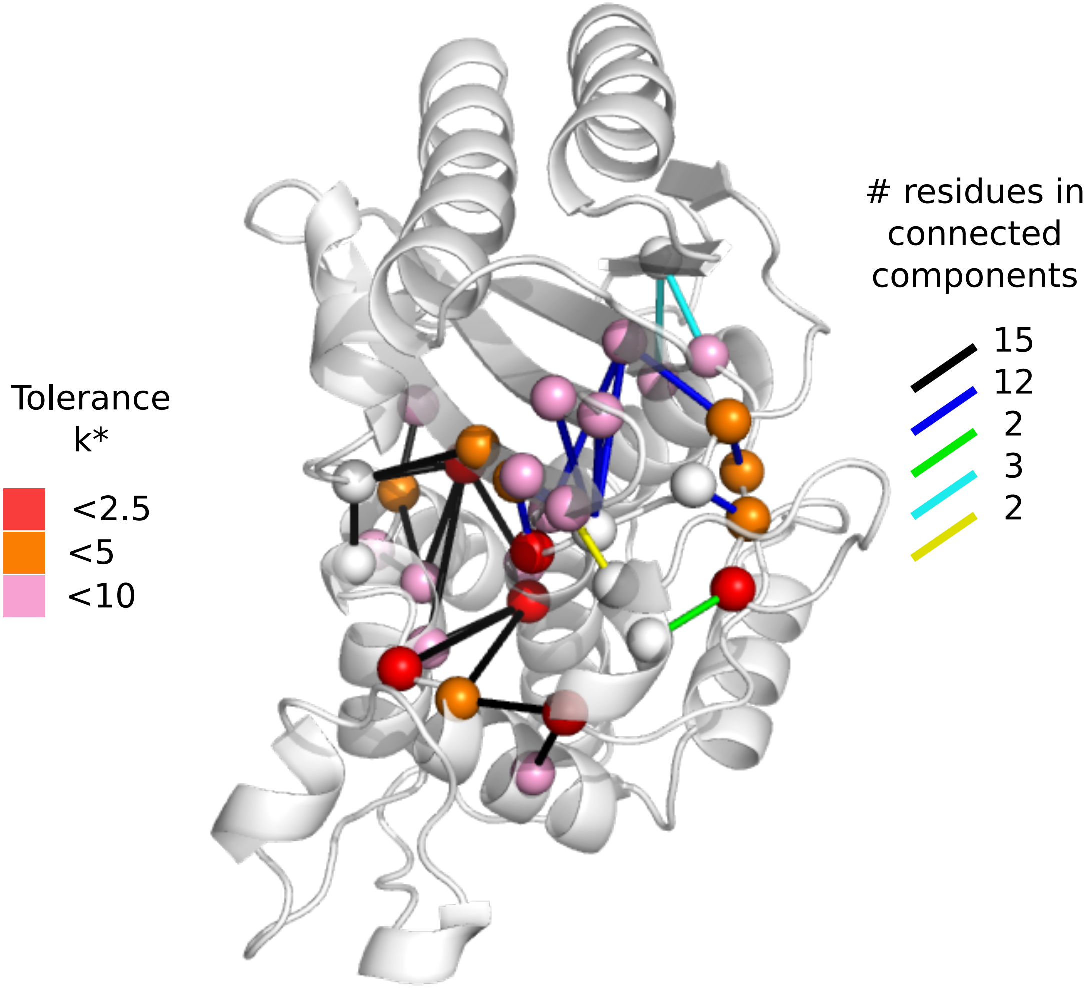

Figure S10. Detection of isolated communication bridges in TEM-1. The residues detected as forming communication bridges are displayed as spheres. The colors of the links between the spheres correspond to different connected components in the network. Spheres are colored according to the experimentally measured tolerance to mutations (in number of tolerated substitutions).

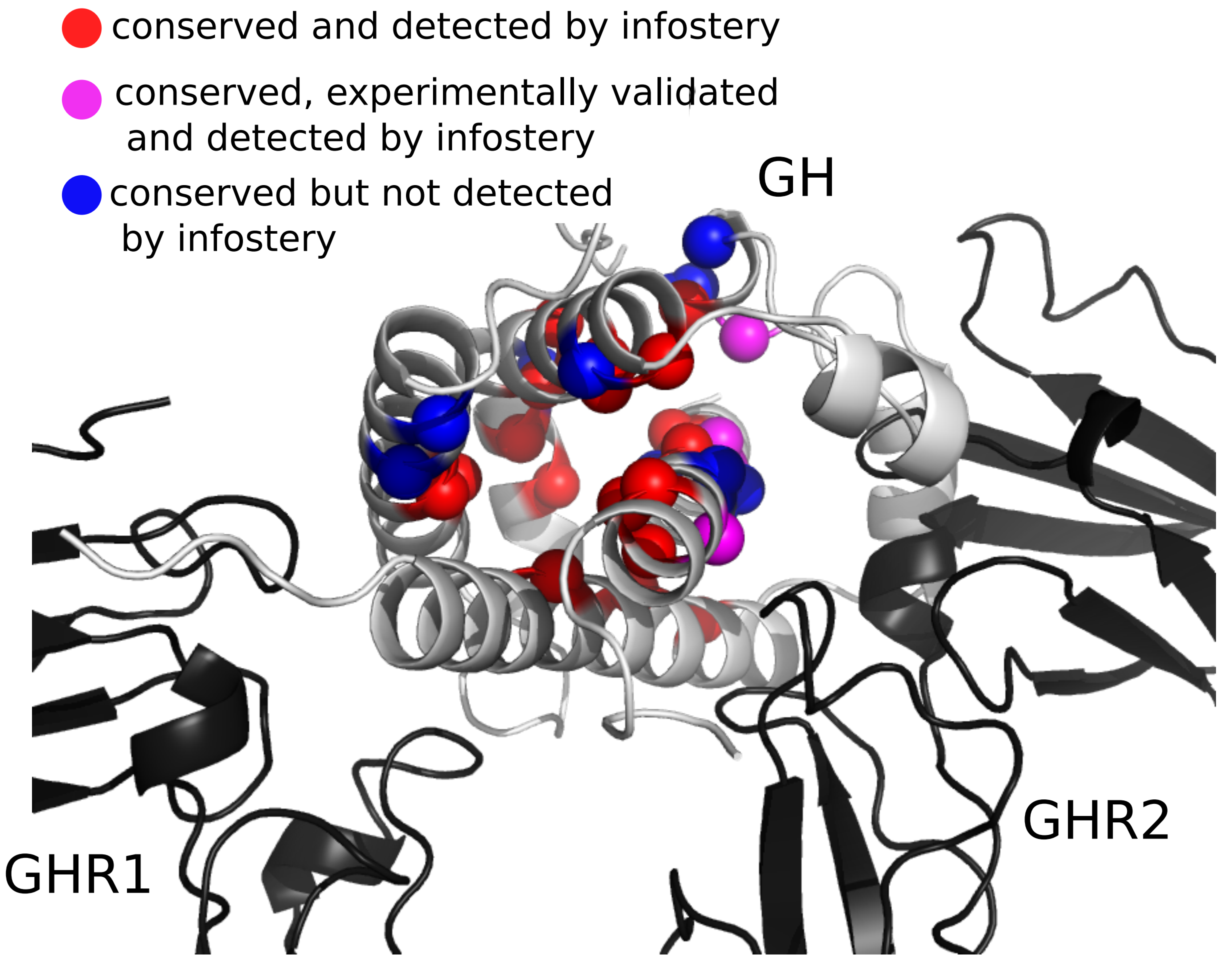

Figure S11. Detection of potentially sensitive positions in growth hormone. The complex between growth hormone (GH, in white) and two monomers of growth hormone receptor (GHR1 and GHR2, in shades of grey) is zoomed in and displayed as a cartoon. Residues from GH protein identified as potentially sensitive to mutations because of their high conservation are highlighted in spheres. Among them, residues detected by using infostery are colored in red. The three residues colored in magenta were experimentally confirmed. They were also detected by infostery.

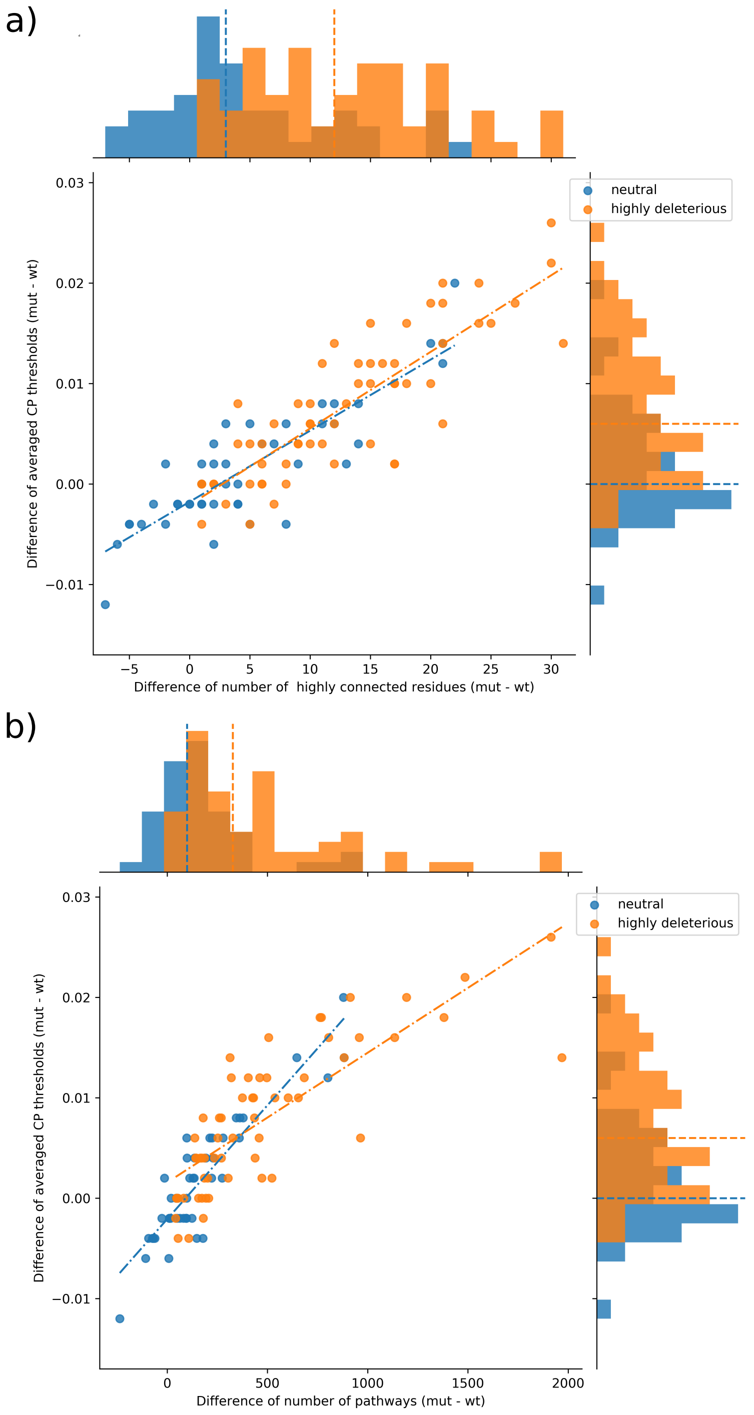

Figure S12. Difference of averaged communication propensity thresholds between mutants and wild-type PSD95^{pdz3}. The difference of averaged communication propensity thresholds (in y-axis) is plotted versus the difference of number of highly connected residues (**a**) or of number of pathways (**b**). The communication propensity threshold is defined based on the backbone communication propensities distribution and is used to decide whether two residues communicate fast or not (see *Materials and Methods* and[8]). The higher the threshold, the less stringent. These threshold values are averaged over the 5 MD replicates, for each system.

References

1. McLaughlin, R. N., Poelwijk, F. J., Raman, A., Gosal, W. S. & Ranganathan, R. The spatial architecture of protein function and adaptation. *Nat.* **491**, 138–142 (2012).
2. Firnberg, E., Labonte, J. W., Gray, J. J. & Ostermeier, M. A comprehensive, high-resolution map of a gene's fitness landscape. *Mol. Biol. Evol.* **31**, 1581–1592 (2014).
3. Cunningham, B. C. & Wells, J. A. Comparison of a structural and a functional epitope. *J. Mol. Biol.* **234**, 554–563 (1993).
4. Besson, A. *et al.* Short stature caused by a biologically inactive mutant growth hormone (GH-C53S). *J. Clin. Endocrinol. Metab.* **90**, 2493–2499 (2005).
5. Sundstrom, M. *et al.* Crystal structure of an antagonist mutant of human growth hormone, G120R, in complex with its receptor at 2.9 A resolution. *J. Biol. Chem.* **271**, 32197–32203 (1996).
6. Doyle, D. A. *et al.* Crystal structures of a complexed and peptide-free membrane protein-binding domain: molecular basis of peptide recognition by PDZ. *Cell* **85**, 1067–1076 (1996).
7. Fonze, E. *et al.* TEM1 beta-lactamase structure solved by molecular replacement and refined structure of the S235A mutant. *Acta Crystallogr. D Biol. Crystallogr.* **51**, 682–694 (1995).
8. Karami, Y., Laine, E. & Carbone, A. Dissecting protein architecture with communication blocks and communicating segment pairs. *BMC Bioinforma.* **17 Suppl 2**, 13 (2016).
